# Risk factors associated with exposure to Crimean-Congo haemorrhagic fever virus in animal workers and cattle, and molecular detection in ticks, South Africa

**DOI:** 10.1371/journal.pntd.0009384

**Published:** 2021-05-28

**Authors:** Veerle Msimang, Jacqueline Weyer, Chantel le Roux, Alan Kemp, Felicity J. Burt, Stefano Tempia, Antoinette Grobbelaar, Naazneen Moolla, Melinda K. Rostal, Whitney Bagge, Claudia Cordel, William B. Karesh, Janusz T. Paweska, Peter N. Thompson

**Affiliations:** 1 Epidemiology Section, Department of Production Animal Studies, Faculty of Veterinary Science, University of Pretoria, Onderstepoort, South Africa; 2 Centre for Emerging Zoonotic and Parasitic Diseases, National Institute for Communicable Diseases of the National Health Laboratory Service, Sandringham, South Africa; 3 Centre for Viral Zoonoses, University of Pretoria, Pretoria, South Africa; 4 Division of Virology, National Health Laboratory Service, Bloemfontein, South Africa; 5 Faculty of Health Sciences, University of the Free State, Bloemfontein, South Africa; 6 School of Public Health, Faculty of Health Sciences, University of the Witwatersrand, Johannesburg, South Africa; 7 EcoHealth Alliance, New York, New York, United States of America; 8 ExecuVet (Pty) LTD, Bloemfontein, South Africa; NIAID Integrated Research Facility, UNITED STATES

## Abstract

Crimean-Congo haemorrhagic fever (CCHF) is a severe tick-borne viral zoonosis endemic to parts of Africa, Europe, the Middle East and Central Asia. Human cases are reported annually in South Africa, with a 25% case fatality rate since the first case was recognized in 1981. We investigated CCHF virus (CCHFV) seroprevalence and risk factors associated with infection in cattle and humans, and the presence of CCHFV in *Hyalomma* spp. ticks in central South Africa in 2017–18. CCHFV IgG seroprevalence was 74.2% (95%CI: 64.2–82.1%) in 700 cattle and 3.9% (95%CI: 2.6–5.8%) in 541 farm and wildlife workers. No veterinary personnel (117) or abattoir workers (382) were seropositive. The prevalence of CCHFV RNA was significantly higher in *Hyalomma truncatum* (1.6%) than in *H*. *rufipes* (0.2%) (*P* = 0.002). Seroprevalence in cattle increased with age and was greater in animals on which ticks were found. Seroprevalence in cattle also showed significant geographic variation. Seroprevalence in humans increased with age and was greater in workers who handled livestock for injection and collection of samples. Our findings support previous evidence of widespread high CCHFV seroprevalence in cattle and show significant occupational exposure amongst farm and wildlife workers. Our seroprevalence estimate suggests that CCHFV infections are five times more frequent than the 215 confirmed CCHF cases diagnosed in South Africa in the last four decades (1981–2019). With many cases undiagnosed, the potential seriousness of CCHF in people, and the lack of an effective vaccine or treatment, there is a need to improve public health awareness, prevention and disease control.

## Author summary

Crimean-Congo haemorrhagic fever (CCHF) is an infection caused by a tick-borne virus endemic to a large geographic area, that is asymptomatic in animals but presents a significant health risk to humans with no effective vaccine. Serological surveys were carried out in rural populations and animals shortly after the first reported case in South Africa in 1981, but little is known about the current seroprevalence of CCHFV in humans and animals, and tick infection rates. We conducted a survey in 2017–18 to obtain recent estimates and examined exposure factors in an area of South Africa where cases are known to occur. We observed a high seroprevalence in cattle of 74.2%. Farm and wildlife workers at risk of occupational exposure to CCHFV had a seroprevalence of 3.9%, which was higher than previously reported. No evidence of CCHFV infection was found in abattoir workers or veterinary professionals in that area. CCHFV RNA was detected in both species of *Hyalomma* ticks but with a higher prevalence (1.6%) in *H*. *truncatum*. This study suggests that CCHFV is widespread in South Africa, that exposure to CCHFV is more common than generally believed in animal-related occupations and that increased public health awareness of risk factors associated with CCHFV infection are required to reduce morbidity and mortality from CCHF.

## Introduction

Crimean-Congo haemorrhagic fever (CCHF) is a tick-borne zoonotic disease of public health importance affecting regions in Africa, Europe, the Middle East and Central Asia [1–3]. The causative agent of CCHF is an RNA virus (CCHFV) of the order Bunyavirales, family *Nairoviridae* and genus *Orthonairovirus* [4]. Hard bodied ticks belonging to the genus *Hyalomma* are the primary vectors of CCHFV [5]. The World Health Organization has prioritized the development of vaccines and therapeutics against CCHF [6–8]; however, no globally approved CCHF vaccine or antiviral treatment are currently available.

Human CCHFV infection is manifested by a rapid onset of fever, severe headache and malaise followed by gastrointestinal symptoms and, in some cases, haemorrhage ranging from petechial rash to pronounced ecchymosis or profuse bleeding. Death occurs in 3–30% of cases due to extensive bleeding, hypovolaemic shock and multi-organ failure [1,9]. CCHFV may also infect a large number of wild and domestic mammals and some avian species, where it rarely causes disease. While animals generally remain ostensibly healthy, high viraemia is briefly produced in infected animals, which can result in infection of feeding ticks and transmission in a vertebrate-tick-vertebrate cycle [10].

CCHFV was first detected in South Africa in 1981 in the North West Province [11]. A field investigation of ticks, sheep and cattle in the area where the case occurred and testing of hares around the country suggested that CCHFV was possibly endemic in South Africa at least four years before the first case was recognized [12]. During 1981–2019, 215 human cases were laboratory confirmed in South Africa, of which 90% originated from the North West, Northern Cape, Free State and Western Cape provinces, with a case fatality rate of 25% [13,14]. Over the past four decades (1981–9, 1990–9, 2000–9, 2010–9) there has been a significant decrease in the sporadic case incidence trend (0.40, 0.26, 0.23, 0.17 per 100,000 rural population, respectively) (S1 Data).

Studies conducted in South Africa in 1986 and 2002 reported that CCHFV seroprevalence in cattle varied by province, from 37.6% in the Northern Cape and a small area in the Western Free State [15], to a mean within-herd seroprevalence of 80%, ranging from 32% to 100% in the North West Province [16]. In 1978–84 [17] and 1986 [15], CCHFV antibodies were detected in 1.5% of farmers and farm workers, and in 1.3% of people who had no contact with confirmed cases located in these provinces. Two seropositive abattoir workers were found amongst purposely selected animal workers in 2017 in the same interior region of South Africa [18].

The current understanding of CCHFV seroprevalence and human exposure in South Africa is limited and is mainly derived from the two farm studies from the 1980s [15,17]. Given the role of vectors, livestock and humans in the maintenance, transmission and impact of CCHFV, a comprehensive understanding requires a One Health approach. In this study we aimed to update available seroprevalence estimates, assess factors associated with CCHFV infection in cattle and animal workers, and estimate the prevalence of CCHFV infection in ticks in areas with historically high number of human cases in South Africa.

## Materials and methods

### Ethics statement

This study was reviewed and approved by the Hummingbird Institutional Review Board, USA (protocol no. 2014–25), DTRA Research Oversight Board, USA (protocol no. CT 2014–33), Witwatersrand University Human Ethics Committee, South Africa (protocol no. 140306) and University of Pretoria Human Research Ethics Committee, South Africa (protocol no. 140/2018). The study was also approved by the Provincial Departments of Health of the Free State and Northern Cape (Ref. no. NC/2015/001). Voluntary and written consent was obtained from all participants included in the study. All persons in farming-, veterinary-, wildlife- or abattoir-related occupational groups aged 12 years or older and who provided consent for participation were eligible for inclusion in the study. Assent with written parental or guardian consent was obtained for study participants aged 12–17 years. Specimen collection from cattle was approved by the Animal Ethics Committee, University of Pretoria, South Africa (protocol no. v020-17 and t005-16), Tufts University, USA (protocol no. G2016-148), USAMRDC Animal Care and Use Review Office, USA (protocol no. CT-2014-33) and the Department of Agriculture, Land Reform and Rural Development, South Africa (protocol no. 12/11/1/1/13). Consent to sample cattle was obtained from all farm owners.

### Study area

**Fig 1:**
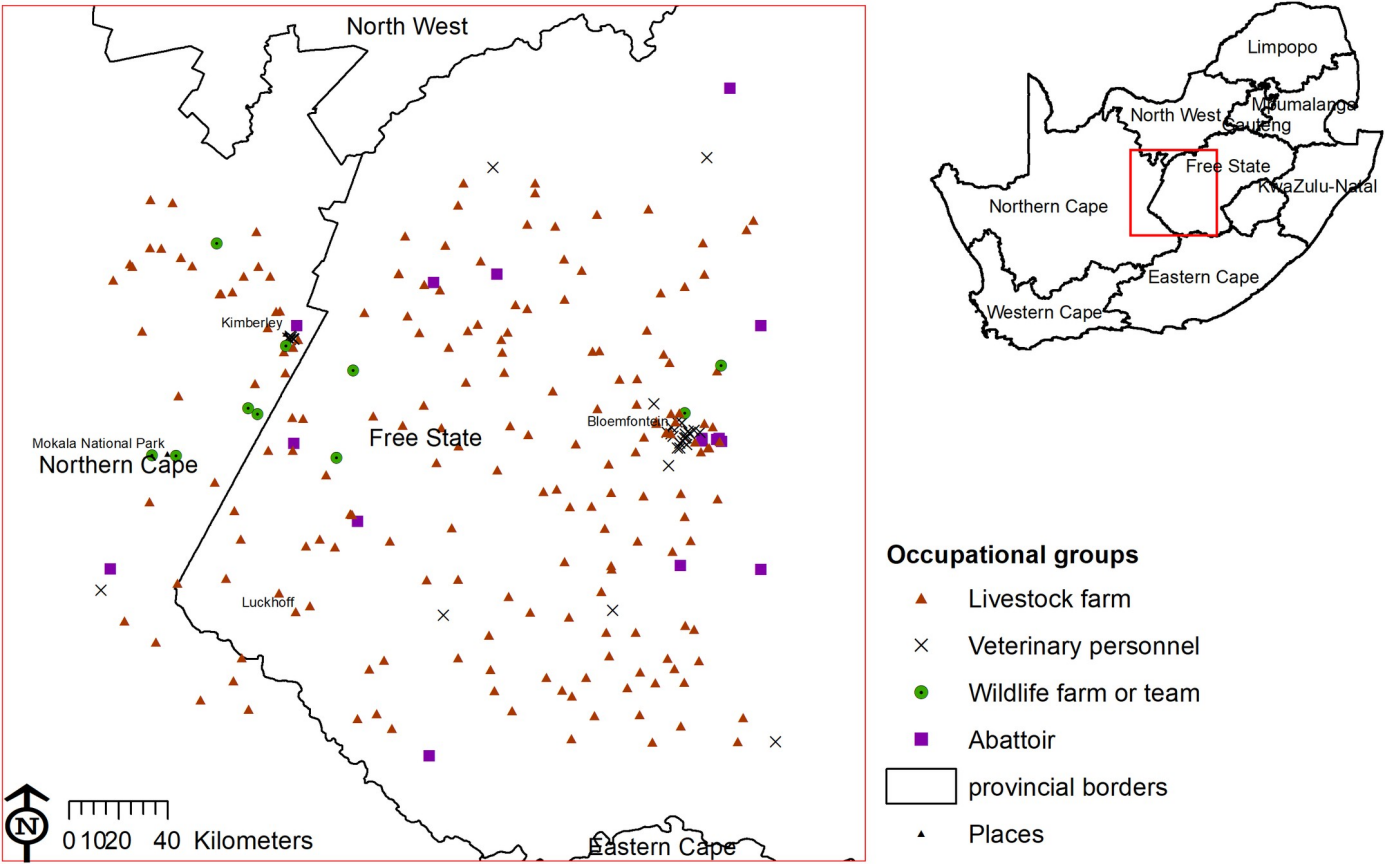
Study area showing human and animal sampling sites for Crimean-Congo haemorrhagic fever virus antibodies or RNA in central South Africa, 2017–18. The map was constructed for the manuscript in Esri ARCGIS 10.2 using country and provincial boundaries from Municipal Demarcation Board [23] and National Geo-Spatial Information [24], coordinates of sampling sites and towns collected during the survey data available under a Creative Commons Attribution (CC BY 4.0) license.

The study area covered ~40,000 km^2^, at 994–1794 m above sea level, in the Free State and Northern Cape provinces, between Bloemfontein (29.0852°S, 26.1596°E) and Mokala National Park (29.1659°S, 24.3197°E) (Fig 1). Most of the study area consists of temperate grassland, classified as Highveld grasslands. The western portion of the study area in Northern Cape falls in the Griqualand West dry savanna, and the southern portion of the study area is part of the Great Karoo. The study area has a cold semi-arid steppe climate, with the exception of the north-western area around Kimberley and the area around Luckhoff which are hot semi-arid steppe [19]. The average winter and summer temperatures are 8°C and 24°C in Bloemfontein and 11°C and 26°C in Kimberley. The area is characterised by long, cold, dry winters and wetter summers. The average humidity in Bloemfontein is 57% versus 31% in Kimberley [20]. The mean annual rainfall ranges between 300–600 mm, increasing from south-west to north-east [21]. While *Hyalomma* spp. ticks are present throughout South Africa, populations are concentrated in the steppe and savanna semi-arid regions [22].

### Study population

The study population comprised people employed in the livestock and game industry within the study area. We estimated there were approximately 40,000 farmers and farm workers living in the study area based on the 2011 National Census data [25], 1300 abattoir workers as estimated from the numbers of employees obtained at each abattoir and 200 veterinary personnel registered with the South African Veterinary Council (SAVC). In the study area, the total number of cattle-owning households was estimated at 4700 using data from the 2011 National Census.

### Inclusion criteria

All persons in the above occupational groups aged 12 years or older and who provided written consent for participation were eligible for inclusion in the study. Assent with parental or guardian consent was obtained for study participants aged 12–17 years. Cattle from farms where people were invited to take part in the survey were included in the study.

### Sampling method

Four cross-sectional surveys were conducted. A One Health farm survey, including farm workers and cattle, was conducted during May-November 2017 (Survey 1). As no comprehensive list of farms was available, farms were randomly selected within the study area using random geographic coordinates generated in ARCGIS 10.2 (Esri, Redlands, CA, USA) and projected on Google Earth (Google LLC, Mountain View, CA, USA). The coordinates were generated with probability proportional to the number of livestock-owning households for each small area of the 2011 National Census [25,26]. The nearest livestock farm to each selected point was approached for participation in the study. If the farm owner declined to participate, we approached the next closest farm to the selected coordinate. Game rangers and capturers from farms where opportunistic wildlife sampling occurred were also invited to participate during October-November 2017 (Survey 2). All veterinary practitioners registered with the SAVC within the study area, as well as all staff of each veterinary practice, were invited to participate in the study carried out from May 2017 to February 2018 (Survey 3). A list of abattoir facilities within or bordering the study region was obtained from the Red Meat Producers Organization. Seventeen of the 28 listed abattoirs were operational and 16 agreed to participate in the study, which was implemented during March-May 2018 (survey 4).

### Sample collection procedure

A maximum of 17 mL of blood was collected from each participant’s cubital vein by a nurse on site. Up to nine cattle per farm were sampled from the jugular or coccygeal veins while restrained in a head gate by the veterinary team. Systematic random sampling was used, or when not possible a combination of haphazard and convenience sampling, to select three apparently healthy animals from each of the following age groups where possible: 6 months to 2 years, 2–4 years and >4 years [26]. *Hyalomma* ticks were collected from their predilection sites on the perineum and the tail tip using thumb forceps and then transferred to a 15 ml pierced vial. The ticks were kept alive in the refrigerator at 4°C in the field and at the National Institute for Communicable Diseases (NICD) laboratory until identification. Blood samples were centrifuged on the day of collection at 1452 g for 8 minutes, after which they were kept at 4°C. Batches were regularly transported to the NICD where sera were transferred into cryovials and frozen (-70°C) until analysis.

### Data collection

Questionnaires were administered to each participant to obtain data on demographics, education, activities, risk behaviours and medical conditions (S1 Text). The education levels used were none, primary (grade 1–7), secondary (grade 8–12), FET = further education & training, HE = higher education. A second questionnaire was administered to the farmer or farm manager to gather population and husbandry data for the cattle (S2 Text). Questionnaires in English, Afrikaans or Sesotho were administered on a tablet using the Open Data Kit application [27]. During the field sampling, herd size and attributes of the cattle such as breed, age category, whether born on the farm or purchased, and the number of ticks found were recorded.

### Diagnostic testing

Human samples were tested for anti-CCHFV IgG using a sandwich enzyme-linked immunosorbent assay (S-ELISA) [28]. As a first step of the S-ELISA, 96-well MaxiSorp microtitre immunoplates (Nunc, Denmark) were coated with primary antibody by adding 100 μl/well of monoclonal mouse anti-CCHF 6E5 diluted 1:2000 in phosphate buffered saline (PBS) pH 7.4. After incubating overnight at 4°C, any unbound antibody was removed by washing. Plates were washed using 3 x 15 second washes with 300 μl of 0.1% of Tween 20 in PBS solution per well. Comparable washes were used between each of the subsequent steps to ensure that only specific CCHFV bound molecules remained attached to the plate. All subsequent incubation periods (with the exception of substrate addition) were performed at 1 h at 37°C in the humidified chamber. All reagents were added to the plate at a volume of 100 μl/well, unless otherwise stated. A blocking protein prepared from the milk protein casein of 10% skimmed milk powder in PBS was then added at 200 μl/well to bind the remaining non-specific protein binding sites in the well. After the plates were incubated and washed, we added antigen diluted 1:500 in diluent buffer made of 2% skimmed milk and PBS to the wells and the primary antibody captured the antigen. Each of the CCHFV (sucrose-acetone extracted antigen prepared from South African isolate) and mock antigens were added to half of the wells [12,28,29]. After incubation and washing, the test and control sera in diluent buffer (1:400) were then added to the wells. Each test serum, negative control serum and conjugate control were tested in duplicate and positive controls were tested in quadruplicate for both CCHF and mock antigen halves. Following further incubation and washing, the secondary antibody, goat anti-human IgG antibodies conjugated with horseradish peroxidase (HRPO) (Invitrogen, Thermo Fisher Scientific, MA, USA), was added as a 1:18000 diluted label enzyme in diluent buffer. A colourless chromogenic substrate, ABTS (2,2’-Azinobis [3-ethylbenzothiazoline-6-sulfonic acid]-diammonium salt) (SeraCare Life Sciences, MA, USA) was added after final incubation and wash, and the enzyme converted it into a coloured end product. The colour intensity of the sample produced by the end product was measured with a spectrophotometer. After 30 minutes leaving the plates at room temperature (22–25°C) in the dark, concentrated stop solution (1% sodium dodecyl sulphate) was added and the optical density of the wells in the plates were read at a wavelength of 405 nm (OD405). The amount of colour produced (measured as absorbance) was directly proportional to the amount of the enzyme, which, in turn, is directly proportional to the amount of antibody that is bound to the antigen captured. The net OD405 values for each sample were determined by subtracting the OD405 detected in wells with mock antigens from wells with CCHFV antigen. OD405 values are absolute measurements, but can be influenced by variables such as temperature. In order to account for the variability, the results were expressed as a function of the control sera reactivity, i.e. a percentage of the positive control serum (PP) using the following formula: $\left(\frac{mean\;net\;OD405\;of\;test\;serum}{mean\;net\;OD405\;of\;positive\;control}x100\right)$. Cut-offs to differentiate between negative and positive samples were as previously determined using known negative samples [28]. The cut-off value was set at 8.4 PP and sera producing PP values <4.2 were considered to be negative. The diagnostic sensitivity was 100% and the specificity 98.7% according to an in-house protocol [28,30–32]. Positive human serum samples were confirmed by repeating the assay on that sample and the overall classification was positive only if both tests were positive. Equivocal results (PP 4.2–8.4%) were retested and the repeated result was recorded. Cattle sera were tested using a similar IgG S-ELISA and positive reactors detected with anti-bovine IgG HRPO conjugate (Invitrogen, Thermo Fisher Scientific, MA, USA) in diluent buffer (1,4000), with PP ≥10 considered positive according to in-house protocol instructions. Sensitivity and specificity of this test in cattle has not been documented [33].

Ticks were systematically washed and dried to allow morphological identification. Ticks were washed using deionised water and identified according to species and sex under a stereo microscope using the taxonomic key by Apanaskevich *et al*, 2008 [34]. Following identification, individual ticks were homogenized in 2 ml screw-cap tubes with a steel bead in Eagle’s minimum essential medium with triple antibiotic solution concentration: penicillin 10,000 IU/mL, streptomycin 10 mg/mL and amphotericin B 25μg/mL. The processed samples were stored in a -20°C freezer until testing.

The tubes were shaken in a tissue lyser at a frequency of 30 Hz per s for 4 min. The samples were centrifuged for 2 min at 6,000 g in a benchtop microcentrifuge to pelletize the debris. A 140 μl aliquot of the supernatant was transferred to a fresh microcentrifuge tube and 560 μl viral lysis (AVL) buffer of a QIAamp viral RNA Mini Qiacube kit (Qiagen, Hilden, Germany) added. After 15 min incubation at room temperature, the samples were loaded onto a QiaCube automated nucleic acid extraction instrument. Samples were eluted in 60 μl AVE buffer, 10 μl of each of 5 eluates were pooled and the presence of CCHFV RNA determined by real time reverse transcription PCR (rtRT-PCR) using the RealStar CCHFV RT-PCR Kit 1.0 (Altona Diagnostics GmbH, Hamburg, Germany) on the Roche LightCycler 480 II Instrument. Pools that tested positive for CCHFV RNA were subsequently analysed individually.

### Data analysis

CCHFV seroprevalence and prevalence in ticks with 95% confidence intervals (CI) were calculated adjusting for clustering using the linearized variance estimator based on a first-order Taylor series linear approximation. Clustering of observations was considered as follows: (i) farms for livestock and game workers, (ii) teams for game capturers, (iii) veterinary clinics for veterinary staff that were not self-employed and (iv) abattoir facilities for slaughter-house workers. For cattle and abattoir studies, estimates were adjusted for unequal sampling fractions by weighting each observation by the sampling weight, calculated as the inverse of the sampling fraction. For the human studies, the apparent seroprevalence was adjusted for test sensitivity and specificity as follows:

$$TP=\frac{\left(AP+Sp-1\right)}{\left(Se+Sp-1\right)}$$

where *TP* = true prevalence, *AP* = apparent prevalence, *Sp* = specificity (98.7%), and *Se* = sensitivity (100%) [35]. Apparent seroprevalence was compared between occupational groups using Fisher’s exact test. To estimate the degree of clustering on the seroprevalence scale, the intraclass correlation coefficient (ICC, ρ) was calculated for cattle and humans as follows:

$$\rho =\frac{\sum_{i=1}^{K}\left\{{\text{Y}}_{\text{i+}}({\text{Y}}_{\text{i+}}-1)-2P({\text{n}}_{\text{i}}-1){\text{Y}}_{\text{i+}}+{\text{n}}_{\text{i}}({\text{n}}_{\text{i}}-1)P^{2}\right\}}{\sum_{i=1}^{K}\left\{{\text{n}}_{\text{i}}({\text{n}}_{\text{i}}-1)P(1-P)\right\}}$$

Where *K* is the number of herds/farms *Y*_*i*+_ is the number of seropositive animals/individuals in the herd/farm *i*, *n*_*i*_ is the number of animals/individuals tested in herd/farm *i* and *P* is the overall (unadjusted) seroprevalence.

In the univariable analysis, we assessed the factors associated with CCHFV seroprevalence in each of the human and cattle populations, using Fisher’s exact test for the analysis among the farm workers and the chi-squared test for the analysis among cattle, and all factors with probability [*P*]-value<0.20 were assessed for inclusion in the multivariable model. Collinearity was assessed among the variables selected for inclusion based on the univariable analysis using pairwise correlation (*r)*, and when |*r*|>0.8, only the most biologically meaningful of the two variables was retained_._

For the multivariable analyses, performed separately for humans and cattle, we used mixed-effects logistic regression models to account for farm clustering. Because of the low CCHFV antibody prevalence in humans, perfect separation sometimes occurred by single covariates or linear combination of variables [36]. Those variables were also omitted from the multivariable model. Livestock farms were sampled near 20 major towns in the study area, thus town was included as another random effect in the analysis. For the farm workers analysis, the towns were grouped in 12 town areas to prevent perfect separation (Barkley-West & Kimberley, Bloemfontein, Boshof & Hertzogville, Brandfort, Bultfontein & Dealesville, Edenburg & Reddersburg & Jagersfontein, Fauresmith, Jakobsdal & Koffiefontein, Luckhoff & Hopetown, Petrusburg, Philippolis, Trompsburg & Smithfield). The final model was obtained using manual backward elimination until all remaining variables were significant (Wald test); however, a variable was retained as a confounder if its removal resulted in a >20% change in the coefficient for another variable considered biologically important in the model. All eliminated variables were then individually re-added in the model and retained if significant [37]. Similarly, sex and species were tested using multiple logistic regression for association with the presence of CCHFV RNA in ticks.

All analyses were done in Stata 13 (StataCorp, College Station, TX, USA) and significance was assessed at *P*<0.05. Maps were created using ARCGIS 10.2 (Esri, Redlands, CA, USA). Farms were defined as seropositive if they had at least one seropositive person (human study) and the within-herd seroprevalence was calculated and mapped (cattle study).

## Results

### Study population

**Fig 2:**
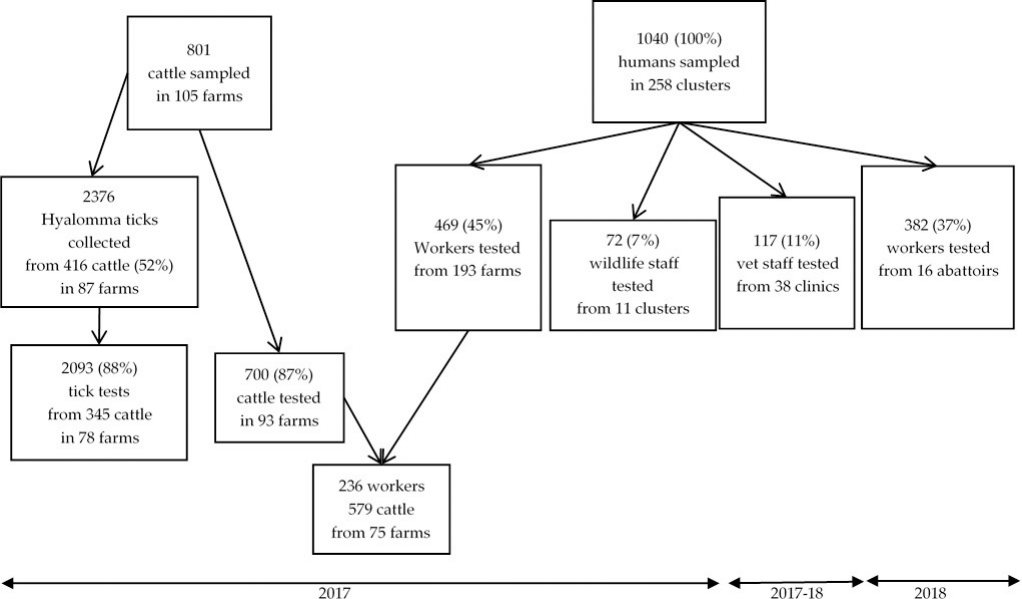
Sampling and testing of humans, cattle and ticks for Crimean-Congo haemorrhagic fever virus antibodies or RNA, central South Africa, 2017–18.

A total of 1040 people participated and were sampled in the 2017–2018 surveys: 469 workers or inhabitants from 193 farms, 117 veterinary professionals from 38 offices and 72 wildlife staff from 11 reserves/offices. In addition, 382 workers from 16 abattoirs participated in the survey conducted in 2018 (Figs 1 and 2). Among the 246 domestic farms visited, serum samples were collected from 801 cattle on 105 farms (43% of total farms visited). A total of 700 cattle sera could be tested for CCHFV IgG. The 801 cattle were examined for *Hyalomma* ticks and a total of 2376 were sampled from 416 cattle on 87 farms. A total of 2093 *Hyalomma* ticks of suitable quality for processing were identified and tested for CCHF RNA from 345 cattle on 78 farms. On 75 farms, both humans and cattle were sampled, which represented 236 humans and 579 cattle (Fig 2).

Farm workers reported completing a lower educational level (secondary) compared to the veterinary, wildlife and abattoir groups (FET–HE). Abattoir workers used personal protective equipment (PPE: overall, apron, boots, hard hat and gloves) most regularly amongst all groups for protection during regular carcass handling: 90.8% in abattoir workers versus 43.6% in farm workers, 46.0% in veterinary workers and 50.7% in wildlife workers. Thirty-seven percent of surveyed farm workers reported irregular use of PPE, mainly limited to overalls and boots. Between 53.3%-67.8% of farm workers, veterinary staff and game workers reported a history of tick bites, whereas many fewer abattoir workers did (24.6%). Abattoir workers had little awareness (7.1%) of tick-associated disease transmission as compared to farm workers (88.0%) and wildlife workers (86.4%) (Table 1).

**Table 1:** Demographic, socio-economic and exposure characteristics of four occupational groups, central South Africa, 2017–2018 (All survey participants, N=1127).

	Characteristic percent (%) or median (interquartile range[IQR]; range)			
Variables	Farm population	Veterinary population	Wildlife workers	Abattoir workers
DEMOGRAPHICS				
Sex				
Male	478/536 (89.2%)	58/125 (46.4%)	71/75 (94.7%)	265/369 (71.8%)
Female	58/536 (10.8%)	67/125 (53.6%)	4/75 (5.3%)	104/369 (28.2%)
Age (years)				
Median (IQR; range)	37 (27–49; 12–85)	36 (29–48; 19–68)	34 (28–41; 21–65)	35 (29–42; 19–77)
Education*				
Median (IQR; range)	Secondary (Primary-FET; None-HE)	HE (FET-HE; Primary(intermediate)-HE)	FET (Secondary-FET; None-HE)	FET (Secondary-FET; None-HE)
EXPOSURE				
Animal contact on a typical day				
<1 h	118/516 (22.9%)	69/115 (60.0%)	21/75 (28.0%)	18/368 (4.9%)
Half-day	223/516 (43.2%)	26/115 (22.6%)	21/75 (28.0%)	34/368 (9.2%)
Entire-day	175/516 (33.9%)	20/115 (17.4%)	33/75 (44.0%)	316/368 (85.9%)
Protective gear used				
Never or rarely	192/514 (37.4%)	10/111 (9.0%)	8/75 (10.7%)	3/368 (0.8%)
Sometimes	98/514 (19.1%)	50/111 (45.0%)	29/75 (38.7%)	31/368 (8.4%)
Regularly or always	224/514 (43.6%)	51/111 (46.0%)	38/75 (50.7%)	334/368 (90.8%)
Bitten by tick in the past				
No	222/515 (43.1%)	37/115 (32.2%)	35/75 (46.7%)	276/366 (75.4%)
Yes	293/515 (56.9%)	78/115 (67.8%)	40/75 (53.3%)	90/366 (24.6%)
Removed ticks from cattle or sheep in the past				
No	379/515 (73.6%)	59/115 (51.3%)	52/75 (69.3%)	300/366 (82.0%)
Yes	136/515 (26.4%)	56/115 (48.7%)	23/75 (30.7%)	66/366 (18.0%)
KNOWLEDGE				
Heard of CCHF				
No	335/515 (65.0%)	-	25/44 (56.8%)	261/369 (70.7%)
Yes	180/515 (35.0%)	-	19/44 (43.2%)	108/369 (29.3%)
Aware that ticks can spread disease				
No	28/515 (5.4%)	-	5/44 (11.4%)	343/369 (93.0%)
Yes	453/515 (88.0%)	-	38/44 (86.4%)	26/369 (7.0%)
Do not know	34/515 (6.6%)	-	1/44 (2.3%)	-

* FET = further education & training, HE = higher education

### Seroprevalence in occupational groups

**Fig 3:**
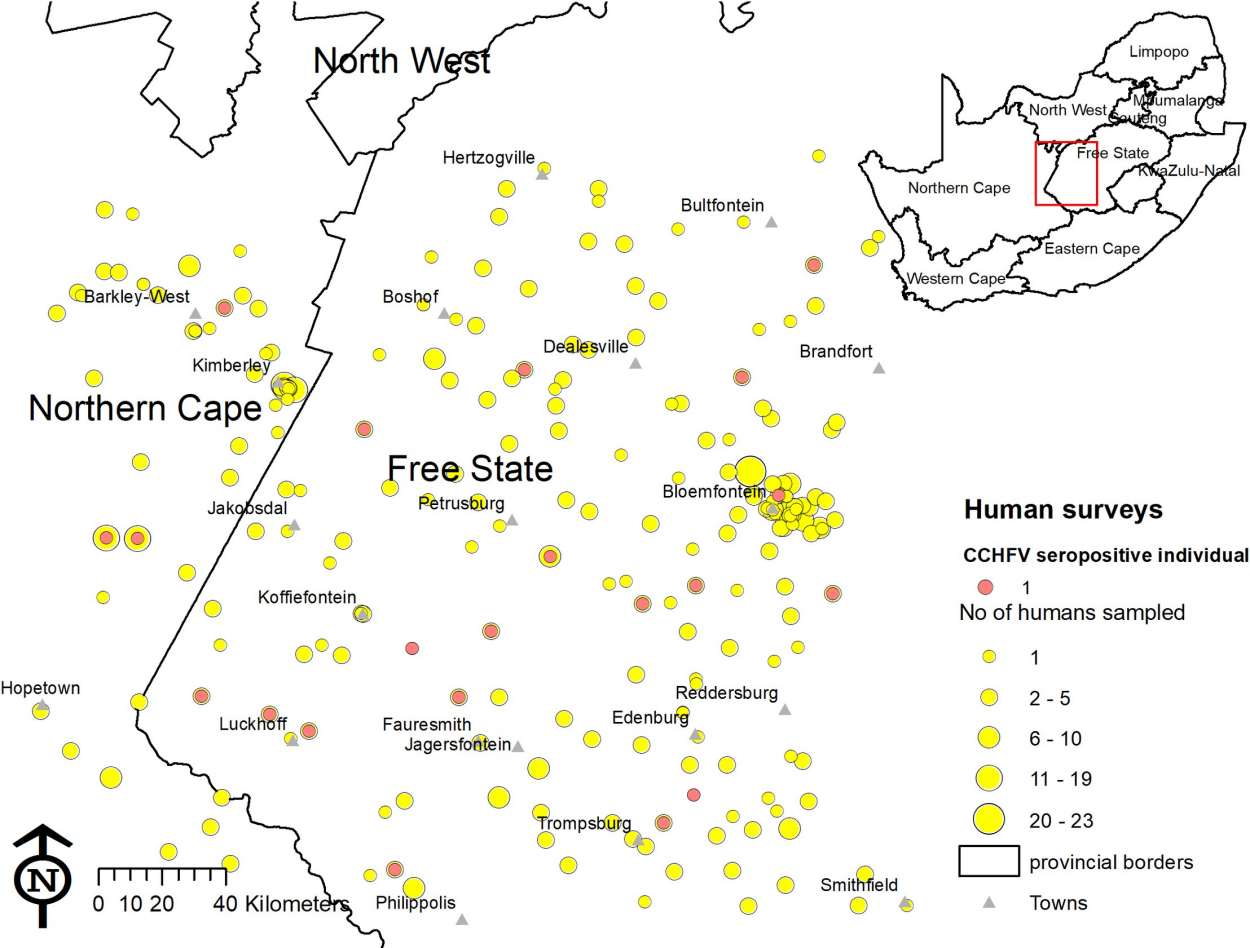
Distribution of individuals seropositive to Crimean-Congo haemorrhagic fever virus among farm workers, veterinary and wildlife personnel in central South Africa, 2017–2018. Yellow circles indicate number of people sampled and red dots show seropositive individuals. The map was constructed for the manuscript in Esri ARCGIS 10.2 using country and provincial boundaries from Municipal Demarcation Board [23] and National Geo-Spatial Information [24], coordinates of sampling sites and towns collected during the survey data available under a Creative Commons Attribution (CC BY 4.0) license.

A total of 1040 human serum samples from 193 domestic animal farms, 38 veterinary clinics, 11 game farms or teams and 16 formal abattoirs were tested. No CCHFV antibodies were detected in abattoir workers or veterinary personnel, while CCHFV antibodies were detected in farm workers and wildlife workers. Overall, 3.8% of farm and 4.2% of wildlife workers were seropositive for CCHFV (Table 2). The farming population was more likely to have been infected than both the abattoir workers *(P*<0.001) and the veterinary personnel (*P* = 0.03). No farms had more than one seropositive person (Fig 3). No seropositive persons were found amongst the 56 female farm workers or the four female wildlife workers.

**Table 2:** Prevalence of antibodies to Crimean-Congo haemorrhagic fever virus by occupational exposure group, central South Africa, 2017–2018.

Exposure group	Year	Clusters	Sample size	No. positive	Seroprevalence % (95%CI)*	Fisher’s exact *P*
Farm workers	2017	193	469	18	3.8 (2.5–6.0) 2.5 (1.2–4.8)**	(reference)
Veterinary personnel	2017–18	38	117	0	0.0 (0.0–0.03)	0.03
Wildlife workers	2017	11	72	3	4.2 (1.3–12.4) 2.9 (0.0–11.3)**	0.75
Abattoir workers	2018	16	382	0	0.0 (0.0–0.01)	<0.001

* Adjusted for clustering;

** True seroprevalence (adjusted for test sensitivity and specificity)

### Factors associated with seroprevalence in farm workers

Ten fixed-effects variables associated (*P*<0.20) with CCHFV seroprevalence on univariable analysis were included in the initial multivariable model and two were retained (Table 3). In the final multivariable model, age was positively associated with the seroprevalence of CCHFV. Participants aged 40–49 years and 50–59 years, had 7.5 (95%CI: 1.9–29.9) and 10.2 (95%CI: 2.4–42.9) times greater odds of being exposed to CCHFV respectively compared to persons aged 12–39 years. One seropositive individual was confirmed positive among the participants aged <18 years. Furthermore, workers who collected samples from or gave injections to animals had higher odds of CCHFV exposure (OR 9.6; 95%CI: 1.3–74.3). Seropositive individuals were distributed across the study area with no clear evidence of clustering (Fig 3); the random effects were non-significant (Table 3) and ICC was 0.

**Table 3:** Factors associated with Crimean-Congo haemorrhagic fever virus exposure in farmers and farm workers in central South Africa, 2017 (S2 Data).

		Univariable analysis	Multivariable analysis	
Variable	CCHFV seropositive n/N (%)	Fisher’s exact *P*-value	Adjusted Odds ratio (95%CI)	*P*-value
**Demographic characteristics**				
**Sex**		0.245		
**Male**	17/406 (4.2%)			
**Female**	0/56 (0.0%)			
Age (years)**		0.001		
**12–39**	3/242(1.2%)		1 (reference)	
**40–49**	7/84 (8.3%)		7.5(1.9–29.9)	0.005
**50–59**	6/52 (11.5%)		10.2(2.4–42.9)	0.002
**≥60**	1/41 (2.4%)		1.8(0.2–17.9)	0.61
**Heard of CCHF**		0.43		
**Yes**	4/161 (2.5%)			
**No**	12/284 (4.2%)			
**Aware that ticks can spread disease**		0.69		
**Yes**	14/396 (3.5%)			
**No**	2/49 (4.1%)			
Education*		0.09		
**None**	4/50 (8.0%)			
**Primary**	5/133 (3.8%)			
**Secondary**	4/109 (3.7%)			
**FET-Higher Education**	3/153 (2.0%)			
**Job**		0.38		
**Farm worker/herdsman/Farm/livestock owner/manager**	15/397 (3.8%)			
**Family, domestic worker, driver**	0/41 (0.0%)			
**Time working with animals**		0.39		
**≤ 2 years**	2/123 (1.6%)			
**>2–8 years**	4/126 (3.2%)			
**>8–17 years**	6/94 (6.4%)			
**> 17 years**	5/113 (4.4%)			
**Owns cattle**		0.44		
**Yes**	13/303 (4.3%)			
**No**	4/156 (2.6%)			
**Owns sheep**		0.62		
**Yes**	12/291 (4.1%)			
**No**	5/168 (3.0%)			
**Owns wildlife**^ʚ^		0.15		
**Yes**	0/60 (0.0%)			
**No**	16/395 (4.1%)			
**Activities in past**				
**Feeding animals**^ʚ^		0.09		
**Yes**	18/391 (4.6%)			
**No**	0/71 (0.0%)			
**Milking cows**		0.32		
**Yes**	12/266 (4.5%)			
**No**	5/196 (2.6%)			
**Herding animals**^ʚ^		0.09		
**Yes**	17/391 (4.3%)			
**No**	0/71 (0.0%)			
**Transporting animals**		0.79		
**Yes**	13/325 (4.0%)			
**No**	4/137 (2.9%)			
**Cleaning animal waste**		0.99		
**Yes**	12/314 (3.8%)			
**No**	5/148 (3.4%)			
Cleaning equipment*		0.11		
**Yes**	15/320 (4.7%)			
**No**	2/142 (1.4%)			
Injection and collection of samples from animals**		0.009		
**Yes**	16/304 (5.3%)		9.6(1.3–74.3)	0.030
**No**	1/158 (0.6%)		1 (reference)	
**Touching blood**		0.99		
**Yes**	12/333 (3.6%)			
**No**	5/129 (3.9%)			
Assisting with animal birthing*		0.13		
**Yes**	14/297 (4.7%)			
**No**	3/165 (1.8%)			
Contact with aborted material*		0.08		
**Yes**	13/251 (5.2%)			
**No**	4/212 (1.9%)			
**Slaughtering of animals**		0.79		
**Yes**	13/327 (4.0%)			
**No**	4/135 (3.0%)			
Burying of dead animals*		0.14		
**Yes**	13/269 (4.8%)			
**No**	4/193 (2.1%)			
Animal contact on a typical day*		0.10		
**< 1 hour**	2/105 (1.9%)			
**Half-day**	12/195 (6.2%)			
**Entire-day**	3/146 (2.1%)			
Taking ticks from hooved animal*		0.16		
**Yes**	7/116 (6.0%)			
**No**	10/ 330 (3.0%)			
**Wearing PPE**		0.61		
**Yes**	8/191 (4.2%)			
**No**	8/253 (3.2%)			
**Bitten by a tick in the past**		0.62		
**Yes**	8/252 (3.2%)			
**No**	8/193 (4.1%)			
**Has chronic medical condition**		0.99		
**Yes**	3/81 (3.7%)			
**No**	15/388 (3.9%)			
**Was hospitalised in past 5 years**		0.78		
**Yes**	5/140 (3.6%)			
**No**	10/312 (3.2%)			
**Type of farm**		0.38		
**Private**	15/366 (4.1%)			
**Communal**	0/36 (0.0%)			
**Altitude of farm**		0.95		
**≤ 1235 meters**	3/99 (3.0%)			
**1236–1306 meters**	5/107 (4.7%)			
**1307–1401 meters**	4/121 (3.3%)			
**≥ 1402 meters**	5/128 (3.9%)			
Goats on farm*		0.03		
**Yes**	1/116 (0.9%)			
**No**	17/289 (5.9%)			
**Sheep on farm**		0.77		
**Yes**	12/297 (4.0%)			
**No**	3/105 (2.9%)			
**Cattle on farm**		0.48		
**Yes**	14/336 (4.2%)			
**No**	1/66 (1.5%)			
**Random Effects**	Variance	SE	95%CI	
**Administrative zones (12 town areas)**	6.01e-35	4.67e-18	-	
**Zones > Farms**	2.27e-32	1.55e-16	-	

*Variable selected for inclusion in multivariable model (Fisher’s exact *P*-value < 0.20);

ʚ Variable not included in multivariable model due to zero count in one category;

** Variable included in final multilevel logistic regression model (*P*-value < 0.05); SE = standard error

### Seroprevalence and related risk factors of cattle exposure

Bonsmara, comprising 39.3% (305) of cattle, was the most common breed and was grouped together with other South African indigenous breeds, Drakensberger, 4.0% (31/777), Nguni, 3.9% (30/777) and Afrikaner, 4.1% (32/777), into the “local breed” category that made up 51.2% (398) of the cattle. Other breeds were grouped as exotic breeds and the breed was unknown for 24 cattle.

**Fig 4:**
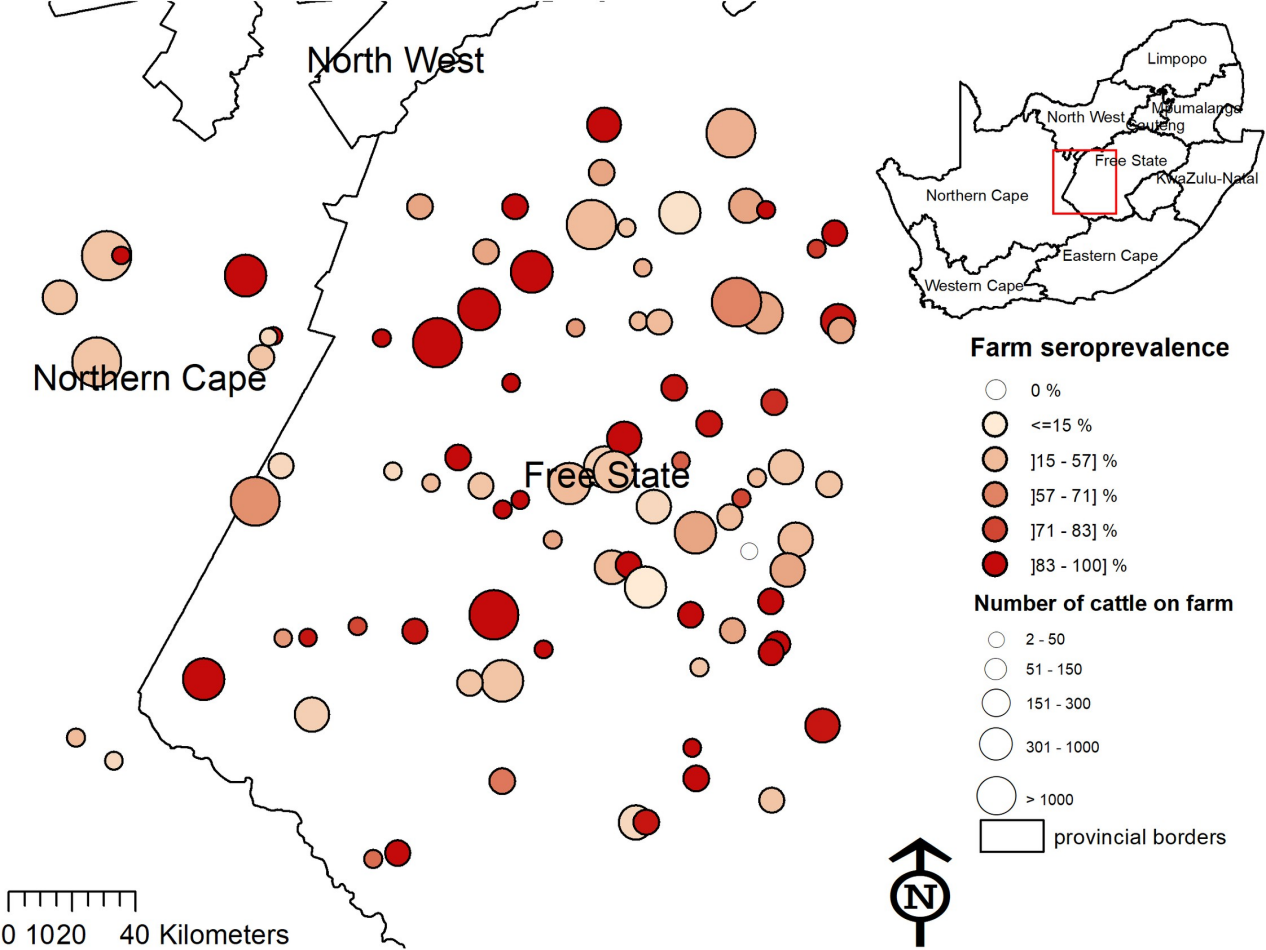
Seroprevalence of Crimean-Congo haemorrhagic fever virus antibodies amongst cattle on farms in Free State and Northern Cape provinces. Size of circle indicates the number of cattle on the farm and colour indicates farm-level seroprevalence. The map was constructed for the manuscript in Esri ARCGIS 10.2 using country and provincial boundaries from Municipal Demarcation Board [23] and National Geo-Spatial Information [24], coordinates of sampling sites and towns collected during the survey data available under a Creative Commons Attribution (CC BY 4.0) license.

Of the 700 cattle sera tested for CCHFV antibodies, 515 were positive, resulting in a crude seroprevalence of 73.6% (95%CI: 70.2–76.7%). After adjustment for clustering and sampling fraction, seroprevalence was estimated to be 74.2% (95%CI: 64.2–82.1%). The median within-herd seroprevalence of 93 herds was 75.0% (IQR: 55.6–100%). The ICC was 0.19. At least one seropositive animal was detected in 92/93 herds (98.9%) (Fig 4).

Fourteen fixed-effect variables were selected for the initial multivariable model and two were retained in the final model (Table 4). The odds of being seropositive increased with age and was 9.2 (95%CI: 4.4–19.1) times higher in cattle 2–4 y old and 30.3 (95%CI: 14.4–64.0) times higher in cattle >4 y old compared to <2 y old. The odds of seropositivity was also higher in cattle with *Hyalomma* ticks found in the perineum or tail brush during the survey (1.7; 95%CI: 1.0–2.8). The seroprevalence varied significantly between administrative zones, ranging from 1/9 (11.1%) to 10/11 (90.9%). The nested random effects of farm and administrative zone were significant (LRT, *P* = 0.0002), indicating significant geographic clustering (Table 4), and the ICC was 0.19.

**Table 4:** Univariable analysis and multivariable model of exposure factors associated with Crimean-Congo haemorrhagic fever virus seroprevalence in cattle in central South Africa, 2017 (S2 Data).

		Univariable analysis	Multivariable analysis	
Variable	CCHFV seropositive n/N (%)	Fisher’s exact *P*-value	Adjusted Odds ratio (95%CI)	*P*-value
**Fixed effects**				
Sex*		<0.001		
**Female**	467/608 (76.8%)			
**Male**	40/81 (49.4%)			
Age (years)**		<0.001		
**<2**	27/99 (27.3%)		1 (reference)	-
**2–4**	130/180 (72.2%)		9.2 (4.4–19.1)	<0.001
**> 4**	338/387 (87.3%)		30.3 (14.4–64.0)	<0.001
Breed*		0.02		
**Exotic**	245/353 (69.4%)			
**Local**	258/332 (77.7%)			
**Cattle born on farm**		0.44		
**Yes**	332/460 (72.2%)			
**No**	145/193 (75.1%)			
Month of sampling*		<0.001		
**Sep**	29/50 (58.0%)			
**Aug**	48/79 (60.8%)			
**Jul**	175/226 (77.4%)			
**Jun**	163/202 (80.7%)			
**May**	94/135 (69.6%)			
Ticks found on cattle**		0.03*		
**Yes**	339/423 (80.1%)		1.7 (1.0–2.8)	0.04
**No**	167/255 (65.5%)		1 (reference)	
Size of farm*		0.14		
**<400 ha**	73/113 (64.6%)			
**400–1000 ha**	130/173 (75.1%)			
**1001–3000 ha**	136/178 (76.4%)			
**>3000 ha**	113/155 (72.9%)			
Altitude of farm*		0.007		
**≤ 1235 meters**	61/102 (59.8%)			
**1236–1306 meters**	126/171 (73.7%)			
**1307–1401 meters**	174/234 (74.4%)			
**≥ 1402 meters**	115/145 (79.3%)			
**Primary animal type on farm**^₻^		<0.001		
**Domestic**	486/668 (72.8%)			
**Wildlife**	8/9 (88.9%)			
**Type of farm**		0.99		
**Private**	461/632 (72.9%)			
**Communal**	33/45 (73.3%)			
Production system*		0.03		
**Feedlot**	43/73 (58.9%)			
**Commercial**	283/378 (74.9%)			
**Semi-commercial**	70/91 (76.9%)			
**Informal**	57/80 (71.3%)			
Main purpose of rearing*		0.19		
**Dairy**	28/43 (65.1%)			
**Meat**	299/414 (72.2%)			
**Wool**	76/94 (80.9%)			
**Mixed purpose**	50/71 (70.4%)			
**Farm exports livestock**		0.48		
**Yes**	78/103 (75.7%)			
**No**	382/528 (72.3%)			
**Animals slaughtered on farm**		0.77		
**Yes**	207/282 (73.4%)			
**No**	246/340 (72.4%)			
Animals kraaled at night*		0.02		
**Kraal with roof**	15/29 (51.7%)	-		
**Open kraal**	66/84 (78.6%)			
**Grazing field**	361/496 (72.8%)			
Rotational grazing*		0.009		
**Yes**	454/616 (73.7%)			
**No**	40/61 (65.6%)			
Contact with animals of other farm*		0.06		
**Yes**	90/135 (66.7%)			
**No**	403/540 (74.6%)			
**Cattle mix with other domestic ruminants**		0.57		
**Yes**	225/313 (71.9%)			
**No**	235/318 (73.9%)			
Quarantine of animals practiced*		0.15		
**Yes**	328/434 (75.6%)			
**No**	85/123 (69.1%)			
Tick control practised on new(born) animals*		0.003		
**Yes**	228/289 (78.9%)			
**No**	258/377 (68.4%)			
**Random Effects**	Variance	SE	(95%CI)	
**Administrative zones (19 towns excl. Smithfield without data)**	0.110	0.229	0.002–6.631	
**Zones > Farms**	0.787	0.353	0.328–1.894	

*Variable selected for inclusion in multivariable model (Fisher’s exact *P*<0.20;

** Variable included in final multilevel logistic regression model (*P* < 0.05); SE = standard error;

₻variables were not included in conventional multivariable model due to collinearity with other variable

### Detection of CCHFV RNA in ticks collected from cattle

Overall, 2376 *Hyalomma* spp. ticks were collected and identified from 416 cattle on 87 farms during the 2017 survey. Sixty-one ticks of other species (34 *Rhipicephalus* spp. and 27 *Margaropus winthemi*) were also gathered from 24 cattle. A median of 1 (IQR: 0–3) *Hyalomma* spp. tick per animal was collected. *Hyalomma rufipes* (n = 1333) was the most frequently found followed by *H*. *truncatum* (n = 1013) and *H*. *glabrum/*hybrid ticks (n = 30); 82% of the *Hyalomma* ticks were male (n/N = 1953/2374). Some tick lysates were insufficient or unusable and therefore not tested. 17 *Hyalomma* spp tested PCR-positive for CCHFV RNA, no CCHFV RNA was detected in the 61 ticks from other genera. Prevalence of CCHFV infection was higher in *H*. *truncatum* (1.6%; 15/914) than in *H*. *rufipes* (0.2%; 2/1149). The odds of detecting CCHFV was 7.9 (95%CI: 2.1–30.1) times greater in *H*. *truncatum* compared to *H*. *rufipes* (*P* = 0.002) and did not differ between male (0.8%, 14/1715) and female (0.8%, 3/376) ticks (*P* = 0.93) (S2 Data).

**Fig 5:**
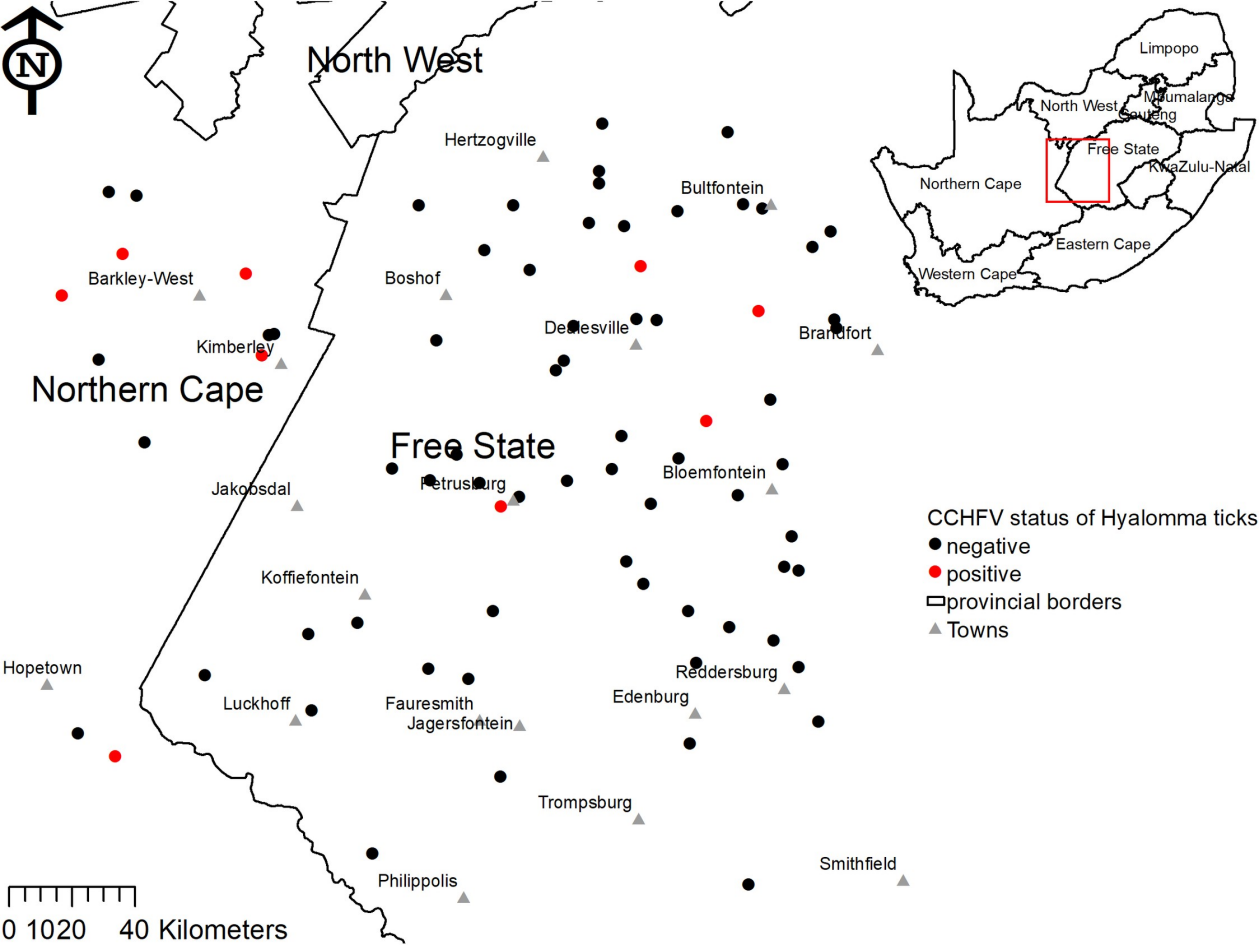
Crimean-Congo haemorrhagic fever virus status amongst cattle *Hyalomma* spp. ticks on farms in Free State and Northern Cape Provinces. The map was constructed for the manuscript in Esri ARCGIS 10.2 using country and provincial boundaries from Municipal Demarcation Board [23] and National Geo-Spatial Information [24], coordinates of sampling sites and towns collected during the survey data available under a Creative Commons Attribution (CC BY 4.0) license.

Crimean-Congo haemorrhagic fever virus infected ticks were detected in all parts of the study area, most frequently in the north-west surrounding Kimberley and Barkly West (Fig 5). The positive ticks were collected from eleven cattle on nine different farms; on these specific cattle the percentage of CCHFV-positive ticks ranged from 5.1% (2/39) to 75.0% (3/4) (Table 5).

**Table 5:** Crimean-Congo haemorrhagic fever virus RNA detection in *Hyalomma* spp. ticks on farms where positive ticks were found in central South Africa, 2017.

Farm	Cattle			Ticks	
	Number on farm	Number sampled for ticks	Number with positive ticks	Sampled from cattle	Number (%) PCR-positive for CCHFV
1	35	9	1	11	1 (9%)
2	30	12	2	39	2 (5%)
3	70	8	1	3	1 (33%)
4	156	3	1	4	3 (75%)
5	97	9	1	6	4 (67%)
6	400	2	2	9	2 (22%)
7	14	5	1	4	2 (50%)
8	15	3	1	10	1 (10%)
9	700	9	1	4	1 (25%)
Total	1517	60	11	90	17 (19%)

## Discussion

Due to limited treatment options, lack of an effective vaccine and high case-fatality rate, CCHFV poses a threat to animal care workers, particularly in the interior plateau of South Africa. The prevalence of exposure to tick-borne zoonotic pathogens, such as CCHFV in humans in Africa, is likely underreported [38–47]. Our series of cross-sectional surveys conducted in different occupational groups, cattle and tick vectors was the most comprehensive study on CCHFV infection and associated risk factors conducted in South Africa and highlights the fact that reported case numbers do not reflect the true level of transmission. The results will aid education of livestock and health care workers in awareness, prevention and management of CCHF.

Our seroprevalence estimate in farm workers of 3.8% was more than double the 1.5% (17/1109) reported from banked samples collected between 1978 and 1984 from 55 farms in the Free State, Northern Cape, and Western Cape [17] and the 1.3% (6/475) reported from individuals sampled in 1986 on 27 farms in the same area as our study [15]. This suggests that human exposure to CCHFV has increased despite the decrease in the number of laboratory-confirmed cases over the past four decades. The seroprevalence estimate suggests that CCHFV infections were several times more frequent than the 215 CCHF human cases reported in the last four decades in South Africa. The results may include a number of individuals with a milder disease than the typical haemorrhagic fever, who did not undergo testing but may still have contributed to the transmission of virus, or possibly even subclinical infection. Therefore, the trend in laboratory-confirmed results should not be used as an accurate indicator of the pattern or extent of human CCHFV infection in South Africa. An increase in seroprevalence over time is possible but must be cautiously interpreted due to geographic variations between the studies, demographics and testing methods, as ELISAs are more sensitive than the immunofluorescence assays used previously and therefore we likely had fewer false negatives [48,49]. Human seroprevalences reported elsewhere in rural Africa ranged from 4.4% in Cameroon in 2005–12 [50] to 13.1% in Senegal in 1989 [51]. We also identified an expected association with seroprevalence and age among the farmers. However, it is interesting to note that the odds of being seropositive drops from 10.2 to 1.8 between the age groups of 50–59 and people aged 60 years or older. It is possible that this indicates waning immunity over time [52,53].

Neither our study nor the 1978–1984 study [17] detected seropositive veterinary professionals, although veterinarians and technicians were amongst laboratory-confirmed cases during 1981–2019. Small animal veterinarians are unlikely to have significant exposure to infected ticks compared to veterinarians working with large herbivores.

Our results provide an estimate of seroprevalence in South African abattoir workers, covering almost all of the formally registered abattoirs across a large area of the highest disease incidence region. Interestingly, our findings show no evidence of previous CCHFV infection in abattoir staff, in contrast to other studies. Two CCHFV seropositive employees (n = 215) at four abattoirs and 30 informal slaughterhouses were detected in a separate slaughterhouse study in 2016–17, but they reported no previous illness resembling CCHF [18] These individuals may have experienced a mild or subclinical form of CCHF. It is also possible that they have their own livestock or were bitten by a tick outside of the abattoir. There is no evidence of less pathogenic strains occurring in SA and differences in severity of disease are more likely dependent on individual hosts. Human exposure to CCHFV has also been detected in abattoir workers in Ghana where 5.7% of workers were seropositive [54]. In addition, South Africa’s biggest reported outbreak of CCHF occurred at an ostrich abattoir in the Western Cape in 1997, evidence of the risk of infection in abattoirs [55]. The lack of exposure detection in abattoir workers in our study could be explained by our finding that abattoir employees were required to wear more personal protective equipment and reported a history of tick bites or removal of ticks from ruminants less frequently compared to participants from the farming sector. This, along with our results, suggests that non-bite exposure among abattoir workers constituted a much lower risk for CCHFV infection. The virus may have been acquired during the Western Cape outbreak by contact with ostrich blood, or lack of understanding of the hazard of CCHFV by crushing ticks while skinning birds. However, abattoirs that participated in our study did not slaughter ostriches; the method of slaughtering and processing, and therefore human exposure, differs between red meat and ostrich abattoirs. Although we did not confirm this, close contact with cattle and ticks are likely human risk factors. Historically, most of the CCHF laboratory-confirmed cases in South Africa have also been associated with tick bites or crushing ticks between one’s fingers [43].

Our analysis also provides evidence of CCHFV infection among wildlife culling teams and those operating in close contact with wildlife. Just one published study included a similar occupational group, 46 hunters in South Africa, although none were seropositive [18]. Wild animals often have a large number of ticks which act as CCHFV amplifiers [10]. Live animal handlers or field workers are at increased risk of CCHFV infection possibly due to blood or tick exposure, but our results did not confirm that people who reported past contact with blood or tick bites had higher seroprevalence. Further, those who reported sampling and administering injections to livestock were more likely to have been exposed, likely because they were in direct contact with cattle, and hence ticks, when performing such tasks.

Our findings showed a high and widespread mean within-herd prevalence of 74% in the Free State and the Northern Cape. CCHFV seroprevalence in cattle was much higher than in humans, likely due to the fact that large herbivores are favoured hosts of adult *Hyalomma* ticks. Our results were consistent with the 80% seroprevalence in cattle in the North West Province in 2002 [16]. However, these estimates were much higher than the 37.6% reported in cattle in 1986 in the Northern Cape and Free State [15]. They were also higher than a serological study in 1983–84, which found that 73% of herds and 26.5% of animals were CCHFV-seropositive. However, this result may not be comparable as it was conducted in cattle from across South Africa including the lowland coastal regions where CCHFV occurs less frequently [17].

Although CCHFV does not cause clinical disease in animals, seroprevalence in cattle is a useful indication of areas with CCHFV circulation. Understanding the farming characteristics and systems associated with higher seroprevalence is important to formulate strategies for reducing infection in or transmission from susceptible cattle, ticks and, subsequently, infection of humans. Farm animals and humans seroconvert at least once and it is generally assumed that they remain immune to CCHFV for 5 years or their lifetime [28,56–58]. During seroconversion, the animal experiences a short viraemia during which feeding ticks can acquire infection or ticks can transmit to each other when co-feeding on non-viraemic animal if they are right next to each other, allowing transmission [10,59]. Our results showed that CCHFV seroprevalence in cattle increased with age, with the majority of animals over two years of age being seropositive. Higher CCHFV seroprevalence in older cattle was also found in various countries [60–62]. Our study identified higher odds of seroprevalence in cattle on which ticks were found. Dipping helps to reduce the load of ticks and potentially the resulting seroprevalence but this effect was not confirmed by our study. Given the longevity of IgG antibodies and the risk of infection if a dipping session is missed, more data are needed to better characterize this effect. Although the overall cattle seroprevalence was high, it was clustered by administrative zone and farm, which suggests geographic variation was driven by factors not measured by our study.

Detection of CCHFV RNA in *Hyalomma* species corroborates the finding of CCHFV in these tick species in an 1983 study [12]. However, differences in CCHFV infection rate between *Hyalomma* spp. in Africa have not previously been described [63]. Our findings suggest exposure to *H*. *truncatum* can pose a greater human risk than *H*. *rufipes* in our study area, a finding that warrants further investigation. The prevalence of infection of ticks was fairly low (<1%) and it may therefore have been missed on many farms. In addition the distribution of ticks is spatially heterogeneous [64,65].

Another potential limitation that may have affected the prevalence of CCHFV detection, was the intensive task of washing and morphologically identifying the ticks, resulting in prolonged storage of the ticks at 4°C. However, we believed that the risk of RNA denaturation was lower for ticks kept in the refrigerator vs implementing multiple freeze-thaw cycles to complete the identifications. A previous study concluded that neither holding temperature (20°C to -70°C) nor period (up to 2 weeks) had an impact on the detection of viral RNA by RT-PCR in mosquito pools [66]. Therefore, we consider that storage at 4°C is unlikely to have significantly affected our results.

Another limitation of the study was that no correlations could be found at farm level between infection in ticks, humans and cattle due to the low sample size per farm, and the low prevalence of seropositive individuals and CCHFV-infected ticks. This limitation of a low sample size also prevented us from conducting a time series analysis to assess whether our seroprevalence levels were affected by seasonal effects on CCHFV and vector prevalence. Further, the study area covered only a part of the area in which CCHFV circulates, and further studies should include larger areas of the Northern Cape and North West provinces. Another limitation was that the study did not include sheep, which also play a role in the distribution of the tick vectors.

## Conclusions

CCHFV is highly prevalent in the inland region of the western Free State and adjacent Northern Cape. Despite the high levels of exposure in cattle in this central region of South Africa, only sporadic cases of CCHF among humans are reported; however, this study shows that human exposure is more frequent than indicated by confirmed cases and the potential for continued outbreaks and subsequent human-to-human spread exists. Infections were most frequent among farm workers who regularly handled domestic and wild hoofstock, possibly because they also had more exposure to tick bites. Further study is warranted on CCHFV infection in ticks and why it was observed more *in Hyalomma truncatum* than in *H*. *rufipes*.

## Supporting information

S1 Data Incidence. (XLSX)

S2 Data Multivardata. (XLSX)

S1 Text Questionnaire participant farmworker survey. (DOCX)

S2 Text Questionnaire private domestic farm survey. (DOC)
