# Supplementary material for: Risk factors associated with exposure to Crimean-Congo haemorrhagic fever virus in animal workers and cattle, and molecular detection in ticks, South Africa
Source: PLoS Negl Trop Dis. 2021 May 28;15(5):e0009384. doi: 10.1371/journal.pntd.0009384 (PMC8162673; doi:10.1371/journal.pntd.0009384)
Supplement: S1 Text — (DOCX) [file pntd.0009384.s003.docx]

Q# indicates both veterinarians and farmer/workers answer the question. -1 indicates that only farmers/workers get the question. -2 indicates only veterinarians get the question. Gray text is not visible to the person taking the survey, but in place to assist with the flow in paper format (vs how it will be given as part of the app on a tablet).

**To be filled out by survey administrator:**

**Admin 1.** Scan the barcode for the associated record,

**Admin 1a**. Enter the barcode manually.

If scanning the barcode is not possible:

**Admin 2.** Farm Identifier:

**Admin 2a.** Farm Name:

**Admin 3.** Date of interview:

**Admin 4.** Collect the current GPS coordinates.

**Admin 4a.** Enter the latitude and longitude manually using the GPS.

If automatic GPS coordinate does not work then:

| Latitude (Degrees) ________ |
| --- |
| Latitude (Minutes) ________ |
| Latitude (Seconds) ________ |
| Longitude (Degrees) ________ |
| Longitude (Minutes) ________ |
| Longitude (Seconds) ________ |

**Admin 5.** Filled in by: _________

**Admin 6.** Interview conducted?  Yes  No

**Admin 7.** Blood sample collected?  Yes  No

**Admin 8.** Does the participant work in veterinary services?  Yes  No

**The remainder of the survey should be filled out by the participant.**

**2.** Gender:  Male  Female

**3.** Race:

Black African

Coloured

White

Indian

Asian

**4.** Please answer one of the following questions:

It is clear in the app that they don’t need to answer both.

How old are you? _______ yrs

Date of birth:

**5-1.** What is your level of education?

No school

Foundation (Gr1-3)

Intermediate (Gr4-6)

Senior (Gr7-9)

FET (Gr10-12)

Higher Education (HE)

**6-1.** Have you heard about animal or human disease outbreaks in the province?  Yes  No

**7-1.** Have you heard of Rift Valley Fever?  Yes  No

**7-1a.** If selected “Yes” to Q7-1 Please indicate the source of this information: *Select all that apply.*

My farm was infected/affected

State Veterinary Services

Radio

Television

Internet

Neighbor/friend's farm was infected/affected

Private Veterinarian

Seminar

Other

If other: Specify other information source.

**7-1b.** If selected “Yes” to Q7-1 Do you know how you catch it?  Yes  No

**8-1.** Have you heard of Congo Fever (Crimean-Congo haemorrhagic fever)?  Yes  No

**8-1a.** If selected “Yes” in Q8-1. Please indicate the source of this information: *Select all that apply*

My farm was infected/affected

State Veterinary Services

Radio

Television

Internet

Neighbour/friend's farm was infected/affected

Private Veterinarian

Seminar

Other

If selected “Other:” in Q8-1a Specify other information source ______

**8-1b.** If selected “Yes” in Q8-1. Do you know how you catch it?  Yes  No

**9-1.** Have you heard of brucellosis (undulant fever/malta fever)?  Yes  No

**9-1a.** If selected “Yes” in Q9-1. Please indicate the source of this information: *Select all that apply*

My farm was infected/affected

State Veterinary Services

Radio

Television

Internet

Neighbour/friend's farm was infected/affected

Private Veterinarian

Seminar

Other

If selected “Other” in Q9-1a: Specify other information source _______

**9-1b.** If selected “Yes” in Q9-1. Do you know how you catch it?  Yes  No

**10-1.** Can you catch a disease in any of the following ways?

Drinking raw milk  Yes  No  I don’t know

Eating raw/uncooked meat  Yes  No  I don’t know

Physical contact with sick animals  Yes  No  I don’t know

Physical contact with dead animals  Yes  No  I don’t know

Physical contact with healthy animals  Yes  No  I don’t know

Bite from animal  Yes  No  I don’t know

Bite from mosquito  Yes  No  I don’t know

Bite from tick  Yes  No  I don’t know

**11-1a.** How long have you worked/lived on a farm? Permit decimals. Answer to one of the questions is required.

*Answer either in years or months.*

Years ____

Months ____

**11-1b.** How long have you been working with farm animals? Permit decimals. Answer to one of the questions is required.

Answer either in years or months.

Years _______

Months _______

**11-1c.** How long have you worked/lived on this farm?

If answer is less than 7 years: At which farm were you employed during 2010-2011?

Name: ___________ Place:_______________

**12.** Do you own livestock? Skip if indicated above that they were a farm or livestock owner  Yes  No

**13.** What species of livestock do you, or have you…? *Flip tablet sideways*

Skip if indicated above that they were a farm owner – that will be captured in the farm questionnaire.

Cattle: Currently own Currently work with Owned at any time in the past Worked with at any time in the past

Sheep: Currently own Currently work with Owned at any time in the past Worked with at any time in the past

Goat: Currently own Currently work with Owned at any time in the past Worked with at any time in the past

Pig: Currently own Currently work with Owned at any time in the past Worked with at any time in the past

Wild animals: Currently own Currently work with Owned at any time in the past Worked with at any time in the past

**13a.** If selected wild animals: What species of wildlife do you, or have you…? *Flip tablet sideways. Please scroll down to view all choices.*

Buffalo Currently own Currently work with Owned at any time in the past Worked with at any time in the past

Springbok Currently own Currently work with Owned at any time in the past Worked with at any time in the past

Blesbok Currently own Currently work with Owned at any time in the past Worked with at any time in the past

Kudu Currently own Currently work with Owned at any time in the past Worked with at any time in the past

Sable antelope Currently own Currently work with Owned at any time in the past Worked with at any time in the past

Roan antelope Currently own Currently work with Owned at any time in the past Worked with at any time in the past

Impala Currently own Currently work with Owned at any time in the past Worked with at any time in the past

Waterbuck Currently own Currently work with Owned at any time in the past Worked with at any time in the past

Reedbuck Currently own Currently work with Owned at any time in the past Worked with at any time in the past

Bontebok Currently own Currently work with Owned at any time in the past Worked with at any time in the past

Hartebeest Currently own Currently work with Owned at any time in the past Worked with at any time in the past

Blue wildebeest Currently own Currently work with Owned at any time in the past Worked with at any time in the past

Black wildebeest Currently own Currently work with Owned at any time in the past Worked with at any time in the past

Eland Currently own Currently work with Owned at any time in the past Worked with at any time in the past

Giraffe Currently own Currently work with Owned at any time in the past Worked with at any time in the past

Zebra Currently own Currently work with Owned at any time in the past Worked with at any time in the past

Tsessebe Currently own Currently work with Owned at any time in the past Worked with at any time in the past

Gemsbok Currently own Currently work with Owned at any time in the past Worked with at any time in the past

Nyala Currently own Currently work with Owned at any time in the past Worked with at any time in the past

Other ruminant Currently own Currently work with Owned at any time in the past Worked with at any time in the past

If other ruminant: Specify other ruminant…

You CURRENTLY own:

You CURRENTLY work with:

You owned AT ANY TIME IN THE PAST:

You worked with AT ANY TIME IN THE PAST:

**14-1.** Have you been involved in any of the following activities with hooved animals (including your own animals)? *Please scroll down to view all choices.*

Feeding Yes, IN THE PAST MONTH Yes, AT ANY TIME IN THE PAST Never

Milking Yes, IN THE PAST MONTH Yes, AT ANY TIME IN THE PAST Never

Herding Yes, IN THE PAST MONTH Yes, AT ANY TIME IN THE PAST Never

Transporting Yes, IN THE PAST MONTH Yes, AT ANY TIME IN THE PAST Never

Cleaning areas with animal waste Yes, IN THE PAST MONTH Yes, AT ANY TIME IN THE PAST Never

Cleaning of equipment used on healthy or sick animals (e.g. dosing gun or hoof trimmers)

Yes, IN THE PAST MONTH Yes, AT ANY TIME IN THE PAST Never

Injecting or collection of specimens from animals

Yes, IN THE PAST MONTH Yes, AT ANY TIME IN THE PAST Never

Touching animal blood Yes, IN THE PAST MONTH Yes, AT ANY TIME IN THE PAST Never

Assisting with the birth of the animals Yes, IN THE PAST MONTH Yes, AT ANY TIME IN THE PAST Never

Contact with aborted foetuses or tissues Yes, IN THE PAST MONTH Yes, AT ANY TIME IN THE PAST Never

Assisting with surgery on animals Yes, IN THE PAST MONTH Yes, AT ANY TIME IN THE PAST Never

Slaughtering/handling of the animal meat Yes, IN THE PAST MONTH Yes, AT ANY TIME IN THE PAST Never

Burying dead animals Yes, IN THE PAST MONTH Yes, AT ANY TIME IN THE PAST Never

Assisting with or performing post-mortem examination of the dead animals

Yes, IN THE PAST MONTH Yes, AT ANY TIME IN THE PAST Never

Other close contact with animals Yes, IN THE PAST MONTH Yes, AT ANY TIME IN THE PAST Never

If other “Yes, IN THE PAST MONTH”: Please explain other contact with animals in the past month:

If other “Yes, AT ANY TIME IN THE PAST”: Please explain other contact with animals AT ANY TIME IN THE PAST:

**15.** On a typical day, how long are you in direct physical contact with hooved animals or their products?

< 1 hour

< 1/2 day

Whole day

**17.** Do you wear protective gear while in physical contact with animals?

Never or rarely

Sometimes

Always

**17-1a** Of the following questions only those pertaining to “yes” answers by the participant in Q14-1a will be asked. None these questions will appear if participants selected “Never or rarely” in Q17.

**17-1.** What type of protective gear do you wear while in performing the following activities: Bring forward only the activities indicated in Q14-1.

Feeding Overall/apron Rubber boots/gumboots Work boots Mask Gloves  Goggles None

Milking Overall/apron Rubber boots/gumboots Work boots Mask Gloves  Goggles None

Herding Overall/apron Rubber boots/gumboots Work boots Mask Gloves  Goggles None

Transporting Overall/apron Rubber boots/gumboots Work boots Mask Gloves  Goggles None

Cleaning areas with animal waste Overall/apron Rubber boots/gumboots Work boots Mask Gloves  Goggles None

Cleaning of equipment used on healthy or sick animals e.g. dosing gun or hoof trimmers Overall/apron Rubber boots/gumboots Work boots Mask Gloves  Goggles None

Injecting or collection of specimens from animals Overall/apron Rubber boots/gumboots Work boots Mask Gloves  Goggles None

Touching animal blood Overall/apron Rubber boots/gumboots Work boots Mask Gloves  Goggles None

Assisting with the birth of the animals Overall/apron Rubber boots/gumboots Work boots Mask Gloves  Goggles None

Contact with aborted foetuses or tissues Overall/apron Rubber boots/gumboots Work boots Mask Gloves  Goggles None

Assisting with surgery on the animals Overall/apron Rubber boots/gumboots Work boots Mask Gloves  Goggles None

Slaughtering/handling of the animal meat Overall/apron Rubber boots/gumboots Work boots Mask Gloves  Goggles None

Burying dead animals Overall/apron Rubber boots/gumboots Work boots Mask Gloves  Goggles None

Assisting with or performing post-mortem examination of the dead animals Overall/apron Rubber boots/gumboots Work boots Mask Gloves  Goggles None

Other close contact with animals Overall/apron Rubber boots/gumboots Work boots Mask Gloves  Goggles None

**17-1a** If the participant indicated they wear mask, gloves or goggles in 17 to following questions will be only for those activities for which the gear was selected.

**How often do you wear your mask when…**

Feeding Always (100%) Most of the time (75-99%) Sometimes (25-75%) Rarely (1-25%)

Milking Always (100%) Most of the time (75-99%) Sometimes (25-75%) Rarely (1-25%)

Herding Always (100%) Most of the time (75-99%) Sometimes (25-75%) Rarely (1-25%)

Transporting Always (100%) Most of the time (75-99%) Sometimes (25-75%) Rarely (1-25%)

Cleaning areas with animal waste Always (100%) Most of the time (75-99%) Sometimes (25-75%) Rarely (1-25%)

Cleaning of equipment used on healthy or sick animals e.g. dosing gun or hoof trimmers Always (100%) Most of the time (75-99%) Sometimes (25-75%) Rarely (1-25%)

Injecting or collection of specimens from animals Always (100%) Most of the time (75-99%) Sometimes (25-75%) Rarely (1-25%)

Touching animal blood Always (100%) Most of the time (75-99%) Sometimes (25-75%) Rarely (1-25%)

Assisting with the birth of the animals Always (100%) Most of the time (75-99%) Sometimes (25-75%) Rarely (1-25%)

Contact with aborted foetuses or tissues Always (100%) Most of the time (75-99%) Sometimes (25-75%) Rarely (1-25%)

Assisting with surgery on the animals Always (100%) Most of the time (75-99%) Sometimes (25-75%) Rarely (1-25%)

Slaughtering/handling of the animal meat Always (100%) Most of the time (75-99%) Sometimes (25-75%) Rarely (1-25%)

Burying dead animals Always (100%) Most of the time (75-99%) Sometimes (25-75%) Rarely (1-25%)

Assisting with or performing post-mortem examination of the dead animals Always (100%) Most of the time (75-99%) Sometimes (25-75%) Rarely (1-25%)

Other close contact with animals Always (100%) Most of the time (75-99%) Sometimes (25-75%) Rarely (1-25%)

**17-1a. How often do you wear your gloves when…**

Feeding Always (100%) Most of the time (75-99%) Sometimes (25-75%) Rarely (1-25%)

Milking Always (100%) Most of the time (75-99%) Sometimes (25-75%) Rarely (1-25%)

Herding Always (100%) Most of the time (75-99%) Sometimes (25-75%) Rarely (1-25%)

Transporting Always (100%) Most of the time (75-99%) Sometimes (25-75%) Rarely (1-25%)

Cleaning areas with animal waste Always (100%) Most of the time (75-99%) Sometimes (25-75%) Rarely (1-25%)

Cleaning of equipment used on healthy or sick animals e.g. dosing gun or hoof trimmers Always (100%) Most of the time (75-99%) Sometimes (25-75%) Rarely (1-25%)

Injecting or collection of specimens from animals Always (100%) Most of the time (75-99%) Sometimes (25-75%) Rarely (1-25%)

Touching animal blood Always (100%) Most of the time (75-99%) Sometimes (25-75%) Rarely (1-25%)

Assisting with the birth of the animals Always (100%) Most of the time (75-99%) Sometimes (25-75%) Rarely (1-25%)

Contact with aborted foetuses or tissues Always (100%) Most of the time (75-99%) Sometimes (25-75%) Rarely (1-25%)

Assisting with surgery on the animals Always (100%) Most of the time (75-99%) Sometimes (25-75%) Rarely (1-25%)

Slaughtering/handling of the animal meat Always (100%) Most of the time (75-99%) Sometimes (25-75%) Rarely (1-25%)

Burying dead animals Always (100%) Most of the time (75-99%) Sometimes (25-75%) Rarely (1-25%)

Assisting with or performing post-mortem examination of the dead animals Always (100%) Most of the time (75-99%) Sometimes (25-75%) Rarely (1-25%)

Other close contact with animals Always (100%) Most of the time (75-99%) Sometimes (25-75%) Rarely (1-25%)

**17-1a. How often do you wear your goggles when…**

Feeding Always (100%) Most of the time (75-99%) Sometimes (25-75%) Rarely (1-25%)

Milking Always (100%) Most of the time (75-99%) Sometimes (25-75%) Rarely (1-25%)

Herding Always (100%) Most of the time (75-99%) Sometimes (25-75%) Rarely (1-25%)

Transporting Always (100%) Most of the time (75-99%) Sometimes (25-75%) Rarely (1-25%)

Cleaning areas with animal waste Always (100%) Most of the time (75-99%) Sometimes (25-75%) Rarely (1-25%)

Cleaning of equipment used on healthy or sick animals e.g. dosing gun or hoof trimmers Always (100%) Most of the time (75-99%) Sometimes (25-75%) Rarely (1-25%)

Injecting or collection of specimens from animals Always (100%) Most of the time (75-99%) Sometimes (25-75%) Rarely (1-25%)

Touching animal blood Always (100%) Most of the time (75-99%) Sometimes (25-75%) Rarely (1-25%)

Assisting with the birth of the animals Always (100%) Most of the time (75-99%) Sometimes (25-75%) Rarely (1-25%)

Contact with aborted foetuses or tissues Always (100%) Most of the time (75-99%) Sometimes (25-75%) Rarely (1-25%)

Assisting with surgery on the animals Always (100%) Most of the time (75-99%) Sometimes (25-75%) Rarely (1-25%)

Slaughtering/handling of the animal meat Always (100%) Most of the time (75-99%) Sometimes (25-75%) Rarely (1-25%)

Burying dead animals Always (100%) Most of the time (75-99%) Sometimes (25-75%) Rarely (1-25%)

Assisting with or performing post-mortem examination of the dead animals Always (100%) Most of the time (75-99%) Sometimes (25-75%) Rarely (1-25%)

Other close contact with animals Always (100%) Most of the time (75-99%) Sometimes (25-75%) Rarely (1-25%)

**17-1a. How often do you wear your coveralls/apron when…**

Feeding Always (100%) Most of the time (75-99%) Sometimes (25-75%) Rarely (1-25%)

Milking Always (100%) Most of the time (75-99%) Sometimes (25-75%) Rarely (1-25%)

Herding Always (100%) Most of the time (75-99%) Sometimes (25-75%) Rarely (1-25%)

Transporting Always (100%) Most of the time (75-99%) Sometimes (25-75%) Rarely (1-25%)

Cleaning areas with animal waste Always (100%) Most of the time (75-99%) Sometimes (25-75%) Rarely (1-25%)

Cleaning of equipment used on healthy or sick animals e.g. dosing gun or hoof trimmers Always (100%) Most of the time (75-99%) Sometimes (25-75%) Rarely (1-25%)

Injecting or collection of specimens from animals Always (100%) Most of the time (75-99%) Sometimes (25-75%) Rarely (1-25%)

Touching animal blood Always (100%) Most of the time (75-99%) Sometimes (25-75%) Rarely (1-25%)

Assisting with the birth of the animals Always (100%) Most of the time (75-99%) Sometimes (25-75%) Rarely (1-25%)

Contact with aborted foetuses or tissues Always (100%) Most of the time (75-99%) Sometimes (25-75%) Rarely (1-25%)

Assisting with surgery on the animals Always (100%) Most of the time (75-99%) Sometimes (25-75%) Rarely (1-25%)

Slaughtering/handling of the animal meat Always (100%) Most of the time (75-99%) Sometimes (25-75%) Rarely (1-25%)

Burying dead animals Always (100%) Most of the time (75-99%) Sometimes (25-75%) Rarely (1-25%)

Assisting with or performing post-mortem examination of the dead animals Always (100%) Most of the time (75-99%) Sometimes (25-75%) Rarely (1-25%)

Other close contact with animals Always (100%) Most of the time (75-99%) Sometimes (25-75%) Rarely (1-25%)

**18.** Do you wash your hands soon after touching animals?  Sometimes  Always  Never or rarely

**18a.** Do you wash your hands: *Select all that apply.* If selected “Sometimes” or “Always” in Q18.

Before touching animals

After touching animals

Between animals

After contact with animal fluids

Before eating

Before going home

After coming home:

For those selected: Percentage time that you wash your hands…

Before touching animals

After touching animal

Between animals

After contact with animal fluids

Before eating

Before going home

After coming home

**18b.** What do you usually wash and dry your hands with? *Select all that apply.*

If selected “Sometimes” or “Always” in Q18.

Water

Soap

Disinfectant handwash

Disposable paper towel

Hand towel

Wipe on your clothes

Air dry

**18c.** What is the source of the hand washing water?

If selected “Water” in Q18b

Tap or hose

From bucket

Animal trough

Pond or pan

River

Other

If other Please specify other hand washing water source, if known: (Not required)

**19-1.** To your knowledge, have you been working with animals (farm or wild) with suspected or confirmed disease IN THE PAST MONTH?

Yes  No

**19-1a.** Which species?

If yes to Q19-1.

Cattle

Sheep

Goat

Pig

Wild animals

**19-1b.** What species of sick wildlife did you work with IN THE PAST MONTH?

If selected “Wild animals” in Q19-1a.

Buffalo

Springbok

Blesbok

Kudu

Sable Antelope

Roan Antelope

Impala

Waterbuck

Reedbuck

Bontebok

Hartebeest

Blue Wildebeest

Black Wildebeest

Eland

Giraffe

Zebra

Tsessebe

Gemsbok

Nyala

Other ruminant:

Specify other ruminant: ______

If selected “Other ruminant” in Q19-1b.

**19-1c.** Describe the infectious disease(s) (suspected or confirmed) and exposure you had? _______

If yes to 19-a.

**19-1d.** To your knowledge, have you been working with animals (farm or wild) with suspected or confirmed disease AT ANY TIME IN THE PAST?

Yes  No

**19-1e.** Which species?

If yes to Q19-1d.

Cattle

Sheep

Goat

Pig

Wild animals

**19-1f.** What species of sick wildlife did you work with AT ANY TIME IN THE PAST?

If selected “Wild animals” in Q19-1e.

Buffalo

Springbok

Blesbok

Kudu

Sable Antelope

Roan Antelope

Impala

Waterbuck

Reedbuck

Bontebok

Hartebeest

Blue Wildebeest

Black Wildebeest

Eland

Giraffe

Zebra

Tsessebe

Gemsbok

Nyala

Other ruminant:

Specify other ruminant: ______

If selected “Other ruminant” in Q19-1f.

**19-1g.** Describe the infectious disease(s) (suspected or confirmed) and exposure you had? ________

If selected “Yes” to 19-1c.

**20-1.** Have you suffered injury from a sharp object while working with the animals?

Yes  No

**20-1a.** If “yes” to 20-1. Please explain the circumstances of the injury and the object: ___________

**21-1.** Do you live within a 20 km radius of a swamp or open water source (other than water pump)?

Yes  No

**21-1a.** Please indicate the approximate distance:

If selected “Yes” for Q21-1, answer to one of the questions is required.

In kilometres _________ OR in minutes walking _________

**22.** Have you had mosquito bites in the past month?  Yes  No  I don’t know

**22a.** Do you get mosquito bites easily?  Yes  No

**22b.** Do you use anything to prevent mosquitoes biting you?  Yes  No

**22c.** What do you use? *Select all that apply.*

If selected “Yes” in Q22b.

Bed nets

Screened windows

Repellent/spray

Other

If selected “Other”: Please list other ways you prevent mosquitoes from biting you: _________

**23.** Have you had a tick bite?  Yes, in the past month  Yes, in the past  No

**24.** Have you taken ticks from hooved animals and squashed them between your fingers?

Yes, in the past month  Yes, in the past  No

**25.** Have you ever eaten meat from a hooved animal found dead?  Yes  No

**25a.** If selected “Yes” in Q25. Specify animal: _______

**25b.** If selected “Yes” in Q25. Was this animal domestic or wild? Domestic Wild

**26.** Do you drink milk?  Yes  No

**26a.** If selected “Yes” in Q26. Is the milk:

Always raw

Always boiled/pasteurized

On occasion raw, other times boiled/pasteurized

**27.** Do you eat rare or not fully cooked meat?  Yes  No

**27a.** If selected “Yes” in Q27. Is the meat:

Always rare/medium rare

Always well cooked

On occasion rare, other times fully cooked

**28.** Have you experienced any of the following symptoms in the...

Fever Last two weeks Last year Neither

Sore joints (more than one, not from work/sport) Last two weeks Last year Neither

Headache Last two weeks Last year Neither

Tiredness (not because you haven’t slept) Last two weeks Last year Neither

Generally unwell Last two weeks Last year Neither

Neck stiffness Last two weeks Last year Neither

Loss of appetite Last two weeks Last year Neither

Nausea, vomiting, stomach cramps Last two weeks Last year Neither

Diarrhea Last two weeks Last year Neither

Sore, red eyes, sensitive to light Last two weeks Last year Neither

Blurred or loss of vision Last two weeks Last year Neither

Confusion/hallucinations Last two weeks Last year Neither

Skin rash Last two weeks Last year Neither

Bleeding (not due to any injury) Last two weeks Last year Neither

Profuse sweats Last two weeks  Last year Neither

Weight loss Last two weeks  Last year Neither

Please specify skin rash you had in the last two weeks: _____________

If selected “Last two weeks” for “Skin rash” in Q28.

Please specify bleeding you had in the last two weeks: _____________

If selected “Last two weeks” for “Bleeding” in Q28.

Please specify skin rash you had in the last year: _____________

If selected “Last year” for “Skin rash” in Q28.

Please specify bleeding you had in the last year: _____________

If selected “Last year” for “Bleeding” in Q28.

**29.** Are you currently on any long-term medication?  Yes  No

If selected “Yes” in Q29: Please specify medication(s): __________

**30.** Did you go to a doctor/clinic/hospital when you were ill with these symptoms?

Yes  No

If selected “Last two weeks” or “Last year” for any symptom in Q28.

If selected “Yes” in Q30.

Please name the health facility and location: _________

What was the diagnosis? _______

**31.** Please indicate any chronic conditions you are treated for: (Select all that apply)

Tuberculosis (TB)

Asthma

Heart disease

Liver disease

Kidney disease

Obesity

Diabetes

Immunosuppression (e.g. HIV, on immunosuppressive medication, malignancy)

Rheumatoid arthritis (debilitating inflammatory joints or deformities)

Other

None

If other: Please describe any health problems you have had in the past year.

__________________________________________________________

**32.** Have you ever had a miscarriage?  Yes  No

Females only.

**33a.** Have you ever been diagnosed with Rift Valley fever?  Yes  No

**33b.** Have you ever been diagnosed with Congo fever?  Yes  No

**33c.** Have you ever been diagnosed with brucellosis?  Yes  No

If selected “Yes” in 33a. Please provide year(s) of diagnoses: __________specify laboratories: __________ *If you do not know, put unknown*

If selected “Yes” in 33b. Please provide year(s) of diagnoses: __________specify laboratories: __________ *If you do not know, put unknown*

If selected “Yes” in 33c. Please provide year(s) of diagnoses: __________specify laboratories: __________ *If you do not know, put unknown*

**34.** Have you ever been hospitalized in the past 10 years?  Yes  No

If yes: What was the reason you were hospitalized?_______________________________________________

If yes: What year were you hospitalized? ____________

**35.** Have you been nursing or been in close contact with a sick person in the past month?  Yes  No

If “Yes” in 35: Specify the illness that person had?

You have successfully completed the survey! Thank you for your participation!

Please hand your device back to the administrator.

Attention survey administrator. Please add any notes here: _______

**Thank you for participating in the survey**
