# Supplementary material for: Risk factors associated with exposure to Crimean-Congo haemorrhagic fever virus in animal workers and cattle, and molecular detection in ticks, South Africa
Source: PLoS Negl Trop Dis. 2021 May 28;15(5):e0009384. doi: 10.1371/journal.pntd.0009384 (PMC8162673; doi:10.1371/journal.pntd.0009384)
Supplement: S2 Text — (DOC) [file pntd.0009384.s004.doc]

**PRIVATE DOMESTIC FARMER QUESTIONNAIRE**

|  |
| --- |

**You are at the start of Farmer Survey. Swipe the screen as shown below to go backward and forward.**

**To be filled out by survey administrator:**

**Admin 1.** Farm Identifier:

**Admin 2.** Farm Name:

**Admin 3.** Date of interview:

­­­­­­­­

**Admin 4.** Collect the current GPS coordinates.

**Admin 4a.** Enter the latitude and longitude manually using the GPS.

If automatic GPS coordinate does not work then:

| Latitude (Degrees) ________ |
| --- |
| Latitude (Minutes) ________ |
| Latitude (Seconds) ________ |
| Longitude (Degrees) ________ |
| Longitude (Minutes) ________ |
| Longitude (Seconds) ________ |

**Admin 5.** Filled in by: _________ *Leave this blank if you are not a survey coordinator.*

**The remainder of the survey should be filled out by the participant.**

Q1 Do you primarily farm domestic or wild animals? Domestic Wildlife

Q2 Is the land your animals are grazed on: Communal Private

Q3 What animal species are/were kept on the farm in the past month (CURRENTLY)? *Select all that apply.*

*Do not include animals that are not owned by the farm. Variants should be listed under the same species.*

Cattle

Sheep

Goat

Pig

Buffalo

Springbok

Blesbok

Kudu

Other

Q3a What animal species are/were kept on the farm in the PAST 12 MONTHS? *Select all that apply.*

*Do not include animals that are not owned by the farm. Variants should be listed under the same species.*

Cattle

Sheep

Goat

Pig

Buffalo

Springbok

Blesbok

Kudu

Other

Q3b Only display relevant questions pertaining to answers in 3 and 3a

Q3b Cattle *If none, enter zero*

What is the ACTUAL number of cattle NOW?

How many of your cattle are < 2 years of age?

How many of your cattle are between 2-4 years of age?

How many or your cattle are >4 years of age?

What is the AVERAGE number of cattle kept on the farm during one month?

Q3b Sheep *If none, enter zero*

What is the ACTUAL number of sheep now?

How many of your sheep are < 2 years of age?

How many of your sheep are between 2-4 years of age?

How many or your sheep are >4 years of age?

What is the AVERAGE number of sheep kept on the farm during one month?

Q3b Goat *If none, enter zero*

What is the ACTUAL number of goats now?

How many of your goats are < 2 years of age?

How many of your goats are between 2-4 years of age?

How many or your goats are >4 years of age?

What is the AVERAGE number of goats kept on the farm during one month?

Q3b Pigs *If none, enter zero*

What is the ACTUAL number of pigs now?

What is the AVERAGE number of pigs kept on the farm during one month?

Q3b Buffalo *If none, enter zero*

What is the ACTUAL number of buffalo now?

What is the AVERAGE number of buffalo kept on the farm during one month?

Q3b Springbok *If none, enter zero*

What is the ACTUAL number of springbok now?

What is the AVERAGE number of springbok kept on the farm during one month?

Q3b Blesbok *If none, enter zero*

What is the ACTUAL number of blesbok now?

What is the AVERAGE number of blesbok kept on the farm during one month?

Q3b Kudu *If none, enter zero*

What is the ACTUAL number of kudu now?

What is the AVERAGE number of kudu kept on the farm during one month?

Q3b Others *If none, enter zero*

List all other species that are/were kept on the farm in the past month (CURRENTLY). Indicate how many of each
species are/were kept on the farm in the past month. *Please separate answers with commas.
Example: 2 horses, 3 camels.*

Q3b List all other species that were kept on the farm in the PAST 12 MONTHS. Indicate the average number of species kept on the farm during one month. *Please separate answers with commas.*

*Example: 2 horses, 3 camels*

Q4 Are any of the farms' animals housed, grazed or allowed to spend time with other species on the farm?

Yes  No

If yes, Which animals are grazed or allowed to spend time with other species on the farm? *Select all that apply.*

Only display relevant choices selected in 3 and 3a

Cattle

Sheep

Goat

Pig

Buffalo

Springbok

Blesbok

Kudu

Other

If other Please list other animals that are grazed or allowed to spend time with other species. *Please separate answers with commas. Examples: horses, camels*

Q4a Only display relevant questions pertaining to answers in 4

Select all animal species on the farm that the cattle mix with. *Select all that apply.*

Sheep

Goat

Pig

Buffalo

Springbok

Blesbok

Kudu

Other

If other What other species do the cattle mix with? *Please separate answers with commas.
 Examples: horses, camels*

Q4a Select all animal species on the farm that the sheep mix with. *Select all that apply.*

Cattle

Goat

Pig

Buffalo

Springbok

Blesbok

Kudu

Other

If other What other species do the sheep mix with? *Please separate answers with commas.
 Examples: horses, camels*

Q4a Select all animal species on the farm that the goats mix with. *Select all that apply.*

Cattle

Sheep

Pig

Buffalo

Springbok

Blesbok

Kudu

Other

If other What other species do the goats mix with? *Please separate answers with commas.
 Examples: horses, camels*

Q4a Select all animal species on the farm that the pigs mix with. *Select all that apply.*

Cattle

Sheep

Goat

Buffalo

Springbok

Blesbok

Kudu

Other

If other What other species do the pigs mix with? *Please separate answers with commas.
 Examples: horses, camels*

Q4a Select all animal species on the farm that the buffalo mix with. *Select all that apply.*

Cattle

Sheep

Goat

Pig

Springbok

Blesbok

Kudu

Other

If other What other species do the buffalo mix with? *Please separate answers with commas.
 Examples: horses, camels*

Q4a Select all animal species on the farm that the springbok mix with. *Select all that apply.*

Cattle

Sheep

Goat

Pig

Buffalo

Blesbok

Kudu

Other

If other What other species do the springbok mix with? *Please separate answers with commas.
 Examples: horses, camels*

Q4a Select all animal species on the farm that the blesbok mix with. *Select all that apply.*

Cattle

Sheep

Goat

Pig

Buffalo

Springbok

Kudu

Other animal

If other What other species do the blesbok mix with? *Please separate answers with commas.
 Examples: horses, camels*

Q4a Select all animal species on the farm that the kudu mix with. *Select all that apply.*

Cattle

Sheep

Goat

Pig

Buffalo

Springbok

Blesbok

Other

If other What other species do the kudu mix with? *Please separate answers with commas.
 Examples: horses, camels*

Please list any additional farm animals interactions not already indicated. *Please separate answers with commas.
 Example: horses mix with camels, ducks mix with chickens*

Q5 Are the farm’s animals grazed or allowed to spend time with wild roaming antelope/hooved animals?

Yes  No

Q5 If yes Which of the farms' animals are grazed or allowed to spend time with wild roaming antelope/hooved animals? *Select all that apply.* Only display relevant choices pertaining to answers in 3 and 3a

Cattle

Sheep

Goat

Pig

Buffalo

Springbok

Blesbok

Kudu

Other

If other Please list any additional farm animals that mix with wild animals that are not already indicated. *Please separate answers with commas. Example: camels mix with steinbuck.*

Q5a Which wild animals (not farmed) graze or spend time with the animals on the farm? *Select all that apply.*

Buffalo

Springbok

Blesbok

Kudu

Sable antelope

Roan antelope

Impala

Waterbuck

Reedbuck

Bontebok

Hartebeest

Blue wildebeest

Black wildebeest

Eland

Giraffe

Zebra

Tsessebe

Gemsbok

Nyala

Other

If other Please list any other wild antelope/hooved animals that spend time with the farm animals. *Please separate answers with commas. Example: farmed steinbuck mix with wild springbok, farmed camels mix with wild springbok.*

Q6 Where is each of your animal species kept during the night? *Select all that apply.*

Only display relevant choices pertaining to answers in 3 and 3a or 3-1 and 3-1a

Cattle

Open kraal

Kraal with roof

Communal kraal

Veld

Other

Q6 Sheep

Open kraal

Kraal with roof

Communal kraal

Veld

Other

Q6 Goat

Open kraal

Kraal with roof

Communal kraal

Veld

Other

Q6 Pig

Open kraal

Kraal with roof

Communal kraal

Veld

Other

Q6 Buffalo

Open kraal

Kraal with roof

Communal kraal

Veld

Other

Q6 Springbok

Open kraal

Kraal with roof

Communal kraal

Veld

Other

Q6 Blesbok

Open kraal

Kraal with roof

Communal kraal

Veld

Other

Q6 Kudu

Open kraal

Kraal with roof

Communal kraal

Veld

Other

Q6 Other

Open kraal

Kraal with roof

Communal kraal

Veld

Other

If other, only options selected in 3 (3-1) and 3a (3a-1) will show up

If other Please list any other location(s) where your cattle are kept during the night.

*Please separate answers with commas.*

If other Please list any other location(s) where your sheep are kept during the night.

*Please separate answers with commas.*

If other Please list any other location(s) where your goats are kept during the night.

*Please separate answers with commas.*

If other Please list any other location(s) where your pigs are kept during the night.

*Please separate answers with commas.*

If other Please list any other location(s) where your buffalo are kept during the night.

*Please separate answers with commas.*

If other Please list any other location(s) where your springbok are kept during the night.

*Please separate answers with commas.*

If other Please list any other location(s) where your blesbok are kept during the night.

*Please separate answers with commas.*

If other Please list any other location(s) where your kudu are kept during the night.

If other Please list any other location(s) where your other animals are kept during the night.

*Please separate answers with commas.*

Q7 Do any animals spend time in the house or compounds occupied by people on the farm? *Refers only to animals*

*you own.*  Yes  No

If yes Which animal species? *Select all that apply.* Only display relevant choices pertaining to answers in 3 and 3a or 3-1 and 3-1a

Cattle

Sheep

Goat

Pig

Buffalo

Springbok

Blesbok

Kudu

Other

If other Please list any other animals that spend time in the house or compounds occupied by people. *Please separate answers with commas. Examples: dogs, cats.*

Q8 Do the farm animals have contact with animals from other farms or owned by different farmers?

Yes  No

Q8a if yes: How often do they have contact in a typical week? *Answer in times per week.*

Q8b if yes: Under which context do they meet? When… *Select all that apply.*

Grazing

Housing

Feeding

Contact through fence

Other

If other: Please list other contexts in which the farm animals have contact with animals from other farms. *Please separate answers with commas.*

Q9 Approximately how many of each does the farm have within access of the animals? *If none, enter zero.*

Water spring(s) / Lake(s) / Natural dam(s) - permanent source

River, stream – constantly flowing source

Pan(s)- seasonal source

Dam(s) - human made source

Water troughs provided

Other outdoor water source

If other: Please describe the other water source(s). *Please separate answers with commas.*

Q9a Water Sources

If Water springs/ lakes/natural dams: On average what percentage of animals on the farm have access to the water spring(s) / Lake(s) / Natural dam(s) (permanent source) at any given time?

Q9a If River, stream - constantly flowing source: On average what percentage of animals on the farm have access to the river, stream - constantly flowing source at any given time?

Q9a If Pan(s) – seasonal source: On average what percentage of animals on the farm have access to the pan(s)- seasonal source at any given time?

Q9a If Dam(s) - human made source: On average what percentage of animals on the farm have access to the dam(s) - human made source at any given time?

Q9a If water troughs: On average what percentage of animals on the farm have access to the water troughs at any given time?

Q9b How many of each of the following water sources have had water available on the farm during the past year? *For example, 10 pans have had water. If none, enter zero.*

Water spring(s) / Lake(s) / Natural dam(s) - permanent source

River, stream – constantly flowing source

Pan(s)- seasonal source

Dam(s) - human made source

Water troughs provided

Other outdoor water source

If other: Please describe the other water source(s). *Please separate answers with commas.*

Q9c What is the longest period of time during which the following water sources have had water during the past year? *For example, Pans on the farm have been flooded for 5 months.* Allow decimals

Water has been available from spring(s) / Lake(s) / Natural dam(s) for:_____ months.

Water has been available from river(s), stream(s) (a constantly flowing source) for: _____ months.

Water has been available from pan(s) (a seasonal source) for:_____ months.

Water has been available from dam(s) or a human made source for:_____ months.

Water has been available from water troughs provided for:_____ months.

Water has been available from other outdoor water source(s) for:_____ months.

If other: Please describe the other water source(s). *Please separate answers with commas.*

Q10 Approximately how far do your animals walk each day to get to the grazing area? *Tap below to answer in km or minutes walking. If kept on the veld put 0.*

Q10a Do you rotate grazing areas for the herds?  Yes  No

Q11 Have you purchased/gained (from gift or birth) animals in the past 12 months?  Yes  No

If yes: Which of the following animals have you purchased/gained (from gift or birth) in the past 12 months?
*Select all that apply.*

Cows

Heifer

Bull

Steer

Calf

Doe goat

Billy goat

Goat kid

Ewe

Ram

Lamb

Sow

Boar

Piglet

Springbok

Blesbok

Kudu

Other

Q11a Asked only for the species indicated as purchased/gained in Q11:

*Approximately how many cows were… If none, enter zero*

| Purchased in the past 12 months |
| --- |
| Given to you in the past 12 months |
| Otherwise gained in the past 12 months |

In what other ways were cows gained in the past 12 months?

Q11a Approximately how many heifers were… *If none, enter zero*

| Purchased in the past 12 months |
| --- |
| Given to you in the past 12 months |
| Otherwise gained in the past 12 months |

In what other ways were heifers gained in the past 12 months?

Q11a Approximately how many bulls were… *If none, enter zero*

| Purchased in the past 12 months |
| --- |
| Given to you in the past 12 months |
| Otherwise gained in the past 12 months |

In what other ways were bulls gained in the past 12 months?

Q11a Approximately how many steers were… *If none, enter zero*

| Purchased in the past 12 months |
| --- |
| Given to you in the past 12 months |
| Otherwise gained in the past 12 months |

In what other ways were steers gained in the past 12 months?

Q11a Approximately how many calves were… *If none, enter zero*

| Purchased in the past 12 months |
| --- |
| Given to you in the past 12 months |
| Born in the past 12 months |
| Otherwise gained in the past 12 months |

In what other ways were calves gained in the past 12 months?

Q11a Approximately how many doe goats were… *If none, enter zero*

| Purchased in the past 12 months |
| --- |
| Given to you in the past 12 months |
| Otherwise gained in the past 12 months |

In what other ways were doe goats gained in the past 12 months?

Q11a Approximately how many billy goats were… *If none, enter zero*

| Purchased in the past 12 months |
| --- |
| Given to you in the past 12 months |
| Otherwise gained in the past 12 months |

In what other ways were billy goats gained in the past 12 months?

Q11a Approximately how many goat kids were… *If none, enter zero*

| Purchased in the past 12 months |
| --- |
| Given to you in the past 12 months |
| Born in the past 12 months |
| Otherwise gained in the past 12 months |

In what other ways were goat kids gained in the past 12 months?

Q11a Approximately how many ewes were… *If none, enter zero*

| Purchased in the past 12 months |
| --- |
| Given to you in the past 12 months |
| Otherwise gained in the past 12 months |

In what other ways were ewes gained in the past 12 months?

Q11a Approximately how many rams were… *If none, enter zero*

| Purchased in the past 12 months |
| --- |
| Given to you in the past 12 months |
| Otherwise gained in the past 12 months |

In what other ways were rams gained in the past 12 months?

Q11a Approximately how many lambs were… *If none, enter zero*

| Purchased in the past 12 months |
| --- |
| Given to you in the past 12 months |
| Born in the past 12 months |
| Otherwise gained in the past 12 months |

In what other ways were lambs gained in the past 12 months?

Q11a Approximately how many sows were… *If none, enter zero*

| Purchased in the past 12 months |
| --- |
| Given to you in the past 12 months |
| Otherwise gained in the past 12 months |

In what other ways were sows gained in the past 12 months?

Q11a Approximately how many boars were… *If none, enter zero*

| Purchased in the past 12 months |
| --- |
| Given to you in the past 12 months |
| Otherwise gained in the past 12 months |

In what other ways were boars gained in the past 12 months?

Q11a Approximately how many piglets were… *If none, enter zero*

| Purchased in the past 12 months |
| --- |
| Given to you in the past 12 months |
| Born in the past 12 months |
| Otherwise gained in the past 12 months |

In what other ways were piglets gained in the past 12 months?

Q11a Approximately how many springbok were… *If none, enter zero*

| Purchased in the past 12 months |
| --- |
| Given to you in the past 12 months |
| Born in the past 12 months |
| Otherwise gained in the past 12 months |

In what other ways were springbok gained in the past 12 months?

Q11a Approximately how many blesbok were… *If none, enter zero*

| Purchased in the past 12 months |
| --- |
| Given to you in the past 12 months |
| Born in the past 12 months |
| Otherwise gained in the past 12 months |

In what other ways were blesbok gained in the past 12 months?

Q11a Approximately how many kudu were… *If none, enter zero*

| Purchased in the past 12 months |
| --- |
| Given to you in the past 12 months |
| Born in the past 12 months |
| Otherwise gained in the past 12 months |

In what other ways were kudu gained in the past 12 months?

List all other species of animals that were gained in the past 12 months. *Please separate with commas.*

Q11a Approximately how many other animals were… *If none, enter zero*

| Purchased in the past 12 months |
| --- |
| Given to you in the past 12 months |
| Born in the past 12 months |
| Otherwise gained in the past 12 months |

In what other ways were other animals gained in the past 12 months?

Q11b If they indicated that they had purchased any animals in Q11:

On average, how many times do you purchase animals during one year? *If none, enter zero. Scroll down to view all.*

Q11b On average, how many… *If none, enter zero. Scroll down to view all.*

| Cows do you purchase at one time? |
| --- |
| Heifers do you purchase at one time? |
| Bulls do you purchase at one time? |
| Steers do you purchase at one time? |
| Calves do you purchase at one time? |
| Doe goats do you purchase at one time? |
| Billy goats do you purchase at one time? |
| Goat kids do you purchase at one time? |
| Ewes do you purchase at one time? |
| Rams do you purchase at one time? |
| Lambs do you purchase at one time? |
| Sows do you purchase at one time? |
| Boars do you purchase at one time? |
| Piglets do you purchase at one time? |
| Springbok do you purchase at one time? |
| Blesbok do you purchase at one time? |
| Kudu do you purchase at one time?  Other animals do you purchase at one time? |

Q11c. Have you sold/lost/slaughtered (ceremonial or for food consumption) animals in the PAST 12 MONTHS?

☐ Yes ☐ No

Q11c Which of the following animals have you sold/lost/slaughtered (ceremonial or for food consumption)?

*Select all that apply.*

Cows

Heifer

Bull

Steer

Calf

Doe goat

Billy goat

Goat kid

Ewe

Ram

Lamb

Sow

Boar

Piglet

Springbok

Blesbok

Kudu

Other

Q11d Asked only for the species indicated in Q11:

Approximately how many cows were… *If none, enter zero*

| Sold in the past 12 months |
| --- |
| Stolen from you in the past 12 months |
| Predated in the past 12 months |
| Died in the past 12 months  Were slaughtered in the past 12 months  otherwise lost in the past 12 months |

In what other ways were cows lost in the past 12 months?

Q11d Approximately how many heifers were… *If none, enter zero*

| Sold in the past 12 months |
| --- |
| Stolen from you in the past 12 months |
| Predated in the past 12 months |
| Died in the past 12 months  Were slaughtered in the past 12 months  otherwise lost in the past 12 months |

In what other ways were heifers lost in the past 12 months?

Q11d Approximately how many bulls were… *If none, enter zero*

| Sold in the past 12 months |
| --- |
| Stolen from you in the past 12 months |
| Predated in the past 12 months |
| Died in the past 12 months  Were slaughtered in the past 12 months  otherwise gained in the past 12 months |

In what other ways were bulls lost in the past 12 months?

Q11d Approximately how many steers were… *If none, enter zero*

| Sold in the past 12 months |
| --- |
| Stolen from you in the past 12 months |
| Predated in the past 12 months |
| Died in the past 12 months  were slaughtered in the past 12 months  otherwise lost in the past 12 months |

In what other ways were steers lost in the past 12 months?

Q11d Approximately how many calves were… *If none, enter zero*

| Sold in the past 12 months |
| --- |
| Stolen from you in the past 12 months |
| Predated in the past 12 months |
| Died in the past 12 months  were slaughtered in the past 12 months  otherwise lost in the past 12 months |

In what other ways were calves lost in the past 12 months?

Q11d Approximately how many doe goats were… *If none, enter zero*

| Sold in the past 12 months |
| --- |
| Stolen from you in the past 12 months |
| Predated in the past 12 months |
| Died in the past 12 months  Were slaughtered in the past 12 months  otherwise lost in the past 12 months |

In what other ways were doe goats lost in the past 12 months?

Q11d Approximately how many billy goats were… *If none, enter zero*

| Sold in the past 12 months |
| --- |
| Stolen from you in the past 12 months |
| Predated in the past 12 months |
| Died in the past 12 months  Were slaughtered in the past 12 months  otherwise lost in the past 12 months |

In what other ways were billy goats lost in the past 12 months?

Q11d Approximately how many goat kids were… *If none, enter zero*

| Sold in the past 12 months |
| --- |
| Stolen from you in the past 12 months |
| Predated in the past 12 months |
| Died in the past 12 months  were slaughtered in the past 12 months  otherwise lost in the past 12 months |

In what other ways were goat kids lost in the past 12 months?

Q11d Approximately how many ewes were… *If none, enter zero*

| Sold in the past 12 months |
| --- |
| Stolen from you in the past 12 months |
| Predated in the past 12 months |
| Died in the past 12 months  Were slaughtered in the past 12 months  Otherwise lost in the past 12 months |

In what other ways were ewes lost in the past 12 months?

Q11d Approximately how many rams were… *If none, enter zero*

| Sold in the past 12 months |
| --- |
| Stolen from you in the past 12 months |
| Predated in the past 12 months |
| Died in the past 12 months  Were slaughtered in the past 12 months  Otherwise lost in the past 12 months |

In what other ways were rams lost in the past 12 months?

Q11d Approximately how many lambs were… *If none, enter zero*

| Sold in the past 12 months |
| --- |
| Stolen from you in the past 12 months |
| Predated in the past 12 months |
| Died in the past 12 months  were slaughtered in the past 12 months  otherwise lost in the past 12 months |

In what other ways were lambs lost in the past 12 months?

Q11d Approximately how many sows were… *If none, enter zero*

| Sold in the past 12 months |
| --- |
| Stolen from you in the past 12 months |
| Predated in the past 12 months |
| Died in the past 12 months  Were slaughtered in the past 12 months  Otherwise lost in the past 12 months |

In what other ways were sows lost in the past 12 months?

Q11d Approximately how many boars were… *If none, enter zero*

| Sold in the past 12 months |
| --- |
| Stolen from you in the past 12 months |
| Predated in the past 12 months |
| Died in the past 12 months  Were slaughtered in the past 12 months  Otherwise lost in the past 12 months |

In what other ways were boars lost in the past 12 months?

Q11d Approximately how many piglets were… *If none, enter zero*

| Sold in the past 12 months |
| --- |
| Stolen from you in the past 12 months |
| Predated in the past 12 months |
| Died in the past 12 months  Were slaughtered in the past 12 months  Otherwise lost in the past 12 months |

In what other ways were piglets lost in the past 12 months?

Q11d Approximately how many springbok were… *If none, enter zero*

| Sold in the past 12 months |
| --- |
| Stolen from you in the past 12 months |
| Predated in the past 12 months |
| Died in the past 12 months  Were slaughtered in the past 12 months  Otherwise lost in the past 12 months |

In what other ways were springbok lost in the past 12 months?

Q11d Approximately how many blesbok were… *If none, enter zero*

| Sold in the past 12 months |
| --- |
| Stolen from you in the past 12 months |
| Predated in the past 12 months |
| Died in the past 12 months  Were slaughtered in the past 12 months  Otherwise lost in the past 12 months |

In what other ways were blesbok lost in the past 12 months?

Q11d Approximately how many kudu were… *If none, enter zero*

| Sold in the past 12 months |
| --- |
| Stolen from you in the past 12 months |
| Predated in the past 12 months |
| Died in the past 12 months  Were slaughtered in the past 12 months  Otherwise lost in the past 12 months |

In what other ways were kudu lost in the past 12 months?

Q11d List all other species of animals that were lost in the past 12 months. *Please separate with commas.*

Approximately how many other animals were… *If none, enter zero. Example: 3 steinbuck, 2 camels.*

| Sold in the past 12 months |
| --- |
| Stolen from you in the past 12 months |
| Predated in the past 12 months |
| Died in the past 12 months  Were slaughtered in the past 12 months  Otherwise lost in the past 12 months |

Q11e Asked if they indicated any species had been sold/lost/slaughtered in Q11

On average, how many times do you sell animals during one year? *If you do not sell animals, enter 0.*

Q11f On average, approximately how many…. *Scroll down to view all.*

| Cows do you sell at one time? |
| --- |
| Heifers do you sell at one time? |
| Bulls do you sell at one time? |
| Steers do you sell at one time? |
| Calves do you sell at one time? |
| Doe goats do you sell at one time? |
| Billy goats do you sell at one time? |
| Goat kids do you sell at one time? |
| Ewes do you sell at one time? |
| Rams do you sell at one time? |
| Lambs do you sell at one time? |
| Sows do you sell at one time? |
| Boars do you sell at one time? |
| Piglets do you sell at one time? |
| Springbok do you sell at one time? |
| Blesbok do you sell at one time? |
| Kudu do you sell at one time? |
| Approximately how many other animals do you sell at one time? |

Q11g If they indicated that they had bought or sold any animals in Q11c: Where did you purchase your animals from OR sell your animals to in the past 12 months? *Select all that apply. Scroll down to view all.*

Auction  Bought  Sold

Other farms/farmers  Bought  Sold

Abattoir  Sold

Other ☐ Bought ☐ Sold

If indicated: How many auctions did you purchase your animals from?

*If you did not purchase any animals at auctions, enter "0"*

How many auctions did you sell your animals at?

*If you did not sell any animals at auctions, enter "0"*

How many other farms did you purchase your animals from?

*If you did not purchase any animals at other farms, enter "0"*

How many other farms did you sell your animals at?

*If you did not sell any animals at other farms, enter "0"*

How many abattoirs did you sell your animals at?

*If you did not sell any animals at abattoirs, enter "0"*

Approximately how far away in Km is the auction where the animal(s) were purchased? *Scroll down to view all.*

Approximately how far away in Km is the second auction where the animal(s) were purchased?

List all other auctions where the animal(s) were purchased and indicate their distance away in Km.

Approximately how far away in Km is the auction where the animal(s) were sold?

Approximately how far away in Km is the second auction where the animal(s) were sold?

List all other auctions where the animal(s) were sold and indicate their distance away in Km.

Approximately how far away in Km is the farm where the animal(s) were purchased? *Scroll down to view all.*

Approximately how far away in Km is the second farm where the animal(s) were purchased?

List all other farms where the animal(s) were purchased and indicate their distance away in Km.

Approximately how far away in Km is the farm where the animal(s) were sold?

Approximately how far away in Km is the second farm where the animal(s) were sold?

List all other farms where the animal(s) were sold and indicate their distance away in Km.

Approximately how far away in Km is the abattoir where the animal(s) were sold? *Scroll down to view all.*

Approximately how far away in Km is the second abattoir where the animal(s) were sold?

List all other abattoirs where the animal(s) were sold and indicate their distance away in Km.

If other: Please specify other places animals were bought or sold.

Q12 What protocols do you implement when introducing new animals into the herd? *Select all that apply.*

Quarantine

Antibiotics

Vaccinations

Dipping/tick treatment

Other

None

If quarantine is selected: How many days are new animals quarantined? *Answer in days*

If other is selected: What other protocols do you implement when introducing new animals into the herd?

Q13 Are the farmed animals or their products intended for international export?  Yes  No

If yes: To where do you export?

Q14 Please estimate the size of the farm in hectares.

Q15 Do you employ people on your farm? ☐ Yes ☐ No

Q15a if yes: Are the workers:  Permanent staff  Seasonal workers

Q15b if permanent selected: How many permanent staff are employed?

Q15b If seasonal selected: How many seasonal staff are employed?

Q16 Do you or any of your employees (and/or families) live on the farm?  Yes  No

If yes: How many

Adults?

Children?

Q17 What is the main industry of the farm?

Meat

Wool (hides)

Dairy

Resale

Dual purpose: meat/dairy

Dual purpose: meat/wool

Tourism/hunting

| Q17a If main industry milk or dual meat/dairy: Is the milk produced on the farm pasteurized/boiled? |
| --- |

Yes  No

| Is raw (non-boiled) milk consumed by anyone on the farm?  Yes  No |
| --- |

Q18 How would you classify your production system?

Feedlot

Commercial (excluding feedlot)

Semi-commercial

Cash Sales

No cash sales

Q19 Who takes care of animals? *Select all that apply.*

Owner

Farm worker

Children

Other

Q19a If owner takes care of animals: What animal specie(s) do(es) the owner take care of? *Select all that apply.*

Cattle

Sheep

Goat

Pig

Buffalo

Springbok

Blesbok

Kudu

Other

If other: What other species does the owner take care of? *Please separate answers with commas. Example: Camels, horses.*

Q19a If farm workers take care of animals: What animal specie(s) do(es) the farm workers take care of? *Select all that* *apply.*

Cattle

Sheep

Goat

Pig

Buffalo

Springbok

Blesbok

Kudu

Other

If other: What other species do the farm workers take care of? *Please separate answers with commas. Example: Camels, horses.*

Q19a If children take care of animals: What animal specie(s) do(es) the children take care of? *Select all that apply.*

Cattle

Sheep

Goat

Pig

Buffalo

Springbok

Blesbok

Kudu

Other

If other: What other species do the children take care of? *Please separate answers with commas. Example: Camels, horses.*

If answered other in Q19 or Q19-1: List all others that take care of animals and indicate the species they take care of. *Example: veterinarians take care of ducks.*

Q20 Are animals slaughtered on the farm?  Yes  No

Q20 If yes to slaughtering: Where are animals slaughtered?

A designated outdoor area for the herd

Owner/worker's home

Local abattoir

Other

Q20a if yes to slaughtering: Who assists in the slaughter?

Owner

Farm workers

Abattoir

Spiritual leader

Local leader

Other

If other: Who else assists with slaughtering? *Please separate answers with commas.*

Q21 Have any animals had an abortion in the past 3 months  Yes  No

Q21a If abortion yes: How many animal species had abortions?

Q21b If abortion yes: Which animal species had abortions?

Cattle

Sheep

Goat

Pig

Buffalo

Springbok

Blesbok

Kudu

Other

Which other animal species had abortions? *Please separate answers with commas. Example: Camels, horses.*

Q21c If yes: Did anyone assist in the handling of the animals or removal of aborted foetus?  Yes  No

Q21d If yes: How many people were involved in assisting with the abortion?

Q21e If yes: Who was involved in assisting with the abortion? *Select all that apply.*

Veterinarian

Owner

Farm worker

Animal health technician

Other

If other: Who else assisted with the abortion?

Q21f If abortion yes: How was the aborted material disposed of? *Select all that apply.*

Buried

*Q22 Please indicate whether you implement any of the following practices on the farm: Scroll down to view all.*

| Do you maintain fencing around the property? | Yes | No |
| --- | --- | --- |
| Do you prevent nose-nose contact between animal species? | Yes | No |
| Do you keep different animal species in different/divided areas on the farm? | Yes | No |
| Do you have separate equipment for different animal species? | Yes | No |
| Do you clean and disinfect equipment used for different herds (same species)? | Yes | No |
| Do you feed, treat, and work with quarantined/sick animal(s) after you are finished with healthy animals? | Yes | No |
| Do you keep pregnant animals separate from herd? | Yes | No |
| Do you clean and disinfect vehicles before and after transporting animals? | Yes | No |
| Do you vaccinate your animals? | Yes | No |
| Do you use tick control (dip animals or give injection) | Yes | No |
| Do you use mosquito/biting fly control? | Yes | No |
| Do you quarantine new animals? | Yes | No |
| Do you use other biosafety practices? | Yes | No |

Q22a If yes to maintain fencing in Q22: What type of fencing do you maintain around property? *Select all that apply.*

Metal/wire

Wood

Electric

Other

If other: What other type of fencing do you maintain around property? *Please separate answers with commas.*

Q22a Please indicate whether you used tick control on cattle in the past 12 months?  Yes  No

Q22a If yes to tick control in Q22: How many times per year do you use tick control on the animals?

Q22a If yes to tick control in Q22: What seasons is the tick control used on the animals?

Summer (December-February)

Fall (March-May)

Winter (June-August)

Spring (September-November)

Q22b If yes to tick control in Winter, How frequently did you use tick control during the winter? *Indicate number of times*

Q22b If yes to tick control in Spring, How frequently did you use tick control during the spring? *Indicate number of times*

Q22b If yes to tick control in Summer, How frequently did you use tick control during the summer? *Indicate number of times*

Q22b If yes to tick control in Fall, How frequently did you use tick control during the fall? *Indicate number of times*

Q22c What method was used? *Select all that apply*

Dipping tank  Hand spraying

Foot bath  Hand dressing

Pour-on  Spray race

Lick in drum with pour-on  Injection

Other

If other: Please list any other method applied. *Please separate answers with commas.*

Q22a If “mosquito/biting fly” control selected in Q22: How many times per year do you use mosquito or fly control?

Q22a If “mosquito/biting fly” control selected in Q22: What mosquito/biting fly control measures do you use? *Please*

*separate answers with commas.*

Q22a If other biosafety measures indicated: Please describe any other biosafety measures you use. *Please separate*

*answers with commas. Example: all in all out.*

Q23 How far away is the closest farm (from the centre of your farm)? *Answer at least 1 of the following:* Km:

OR minutes drive:

OR minutes walking:

Q24 Has brucellosis been confirmed on your farm at any time in the past?  Yes  No

If yes: Which animals were infected? *Select all that apply.*

Cattle

Sheep

Goat

Pig

Buffalo

Springbok

Blesbok

Kudu

Other

If other: What other animal species were confirmed having brucellosis? *Please separate answers with commas. Example: Camels, horses*

Q24a If yes to Q24 or Q24-1 or Q24-2: When was brucellosis confirmed? *Enter the* *month and year.*

Enter Date

Month (Enter 1-12)

Year

Q24b Has there been any suspicion of brucellosis on the farm in the past 3 months?  Yes  No

Q24c Has Rift Valley fever been confirmed on your farm at any time in the past?  Yes  No

If yes: Did a veterinarian confirm the diagnosis?

Yes, a state veterinarian  Yes, a private veterinarian  No

If yes: Which animals were infected? *Select all that apply.*

Cattle

Sheep

Goat

Pig

Buffalo

Springbok

Blesbok

Kudu

Other

If other: What other animal species were confirmed having Rift Valley fever? *Please separate answers with commas. Example: Camels, horses.*

For each animal infected in 24c: Approximately how many animals from each species died from Rift Valley fever during the 2010-2011 outbreak that affected your farm? *If none, enter 0.*

Adult cattle ______

Calves ______

Adult sheep ______

Lambs ______

Adult goats ______

Goat kids ______

Pig ______

Buffalo ______

Springbok ______

Blesbok ______

Kudu ______

Other ______

For each animal infected in 24c: Approximately how many animals from each species aborted due to Rift Valley fever during the 2010-2011 outbreak that affected your farm? *If none, enter 0.*

Cows ______

Ewes ______

Lambs ______

Does ______

Pigs ______

Buffalo ______

Springbok ______

Blesbok ______

Kudu ______

Other ______

Q24d If yes to Q24c: When was Rift Valley fever confirmed? *Enter the month and year.*

Enter Date

Month (Enter 1-12)

Year

Q24e Has there been any suspicion of Rift Valley fever on the farm in the past 3 months?  Yes  No

Q25 Have any animals been vaccinated for brucellosis?  Yes  No

If yes: Which animals were vaccinated? *Select all that apply.*

Cattle

Sheep

Goat

Pig

Buffalo

Springbok

Blesbok

Kudu

Other

If yes: What other animals have been vaccinated for brucellosis? *Please separate answers with commas. Example: Camels, horses.*

If yes: When was the most recent vaccine given? *Enter the month and year.*

Enter Date

Month (Enter 1-12)

Year

If yes: What percentage of animals currently on the farm are vaccinated?

If yes: Which vaccine(s) were used?

RB51

S19

RB51 and S19

Other

If other: Please list any other vaccine given. *Please separate answers with commas.*

Q25a Have any animals been vaccinated for Rift Valley fever?  Yes  No

If yes: Which animals were vaccinated? *Select all that apply.*

Cattle

Sheep

Goat

Pig

Buffalo

Springbok

Blesbok

Kudu

Other

If yes: What other animals have been vaccinated for Rift Valley fever? *Please separate answers with commas. Example: Camels, horses.*

If yes: When was the most recent vaccine given? *Enter the month and year.*

Enter Date

Month (Enter 1-12)

Year

If yes: What is your protocol for Rift Valley fever vaccination on your farm?

Once for all young

Every year

Only before/during outbreaks

Never

If yes: What percentage of animals currently on the farm are vaccinated?

If yes: Which vaccine(s) were used?

Smithburn

Clone 13

Inactivated

Clone 13 and inactivated

modified-live

Unknown

Other

If other: Please list any other vaccine given. *Please separate answers with commas.*

**You have successfully completed the survey! Thank you for your participation!**

**Please hand your device back to the administrator**

**Attention Survey Administrators: Please add any notes here**
